# *N*‑Acetylcysteine-Capped TLQP21-Containing Au Nanocages Alleviate Depression in Mice

**DOI:** 10.1021/acsnano.5c11681

**Published:** 2025-10-14

**Authors:** Meng Shi, Xiangyu Li, Zhen Fan, Yi Wang, Congcong Li, Yuanmeng Ning, Yizhao Ma, Min Sun, Xiaohuan Xia, Jianzhong Du, Jialin C. Zheng

**Affiliations:** † Center for Translational Neurodegeneration and Regenerative Therapy, 592018 Tongji Hospital Affiliated to Tongji University School of Medicine, Shanghai 200065, China; ‡ Shanghai Key Laboratory of Anesthesiology and Brain Functional Modulation, Clinical Research Center for Anesthesiology and Perioperative Medicine, Translational Research Institute of Brain and Brain-Like Intelligence, Shanghai Fourth People’s Hospital, School of Medicine, 12476 Tongji University, Shanghai 200434, China; § Department of Polymeric Materials, School of Materials Science and Engineering, Tongji University, Shanghai 201804, China; ∥ Translational Research Center, Shanghai Yangzhi Rehabilitation Hospital Affiliated to Tongji University School of Medicine, Shanghai 201619, China; ⊥ School of Materials Science and Engineering, East China University of Science and Technology, 130 Meilong Road, Shanghai 200237, China; # State Key Laboratory of Cardiology and Medical Innovation Center, Shanghai East Hospital, School of Medicine, Tongji University, Shanghai 200120, China; ¶ Innovation Center of Medical Basic Research for Brain Aging and Associated Diseases, Ministry of Education, Tongji University, Shanghai 200330, China; ∇ Collaborative Innovation Center for Brain Science, Tongji University, Shanghai 200072, China

**Keywords:** major depressive disorder, Au nanocages, TLQP21, NAC, chronic unpredictable mild stress

## Abstract

Major depressive disorder (MDD) is the most prevalent neuropsychiatric disorder globally. Promising therapies for MDD are urgently needed due to the limited effectiveness, delayed efficacy, and non-negligible side effects of current treatments. Oxidative stress and neuroinflammation have been recognized as key contributors to MDD. Here, we developed an antioxidant *N*-acetylcysteine (NAC)-capped Au nanocage (TNNC) that entrapped VGF-derived peptide TLQP21 with neuro-immunomodulatory effects. Once internalized by cells suffering from oxidative stress, NAC was consumed, and TLQP21 was released from TNNC. TNNC administration alleviated MDD-like behaviors of the chronic unpredictable mild stress (CUMS)-exposed mice and effectively relieved oxidative stress in the brains. Moreover, TLQP21 in TNNC inhibits the activation, excessive synaptic pruning, and inflammatory responses of microglia through targeting the complement C1q receptor (C1qR) and complement C3a receptor 1 (C3aR1). This work provides a bioinspired strategy to target multiple pathogenic factors in one nanoparticle for the intervention of MDD and other diseases.

## Graphical abstract

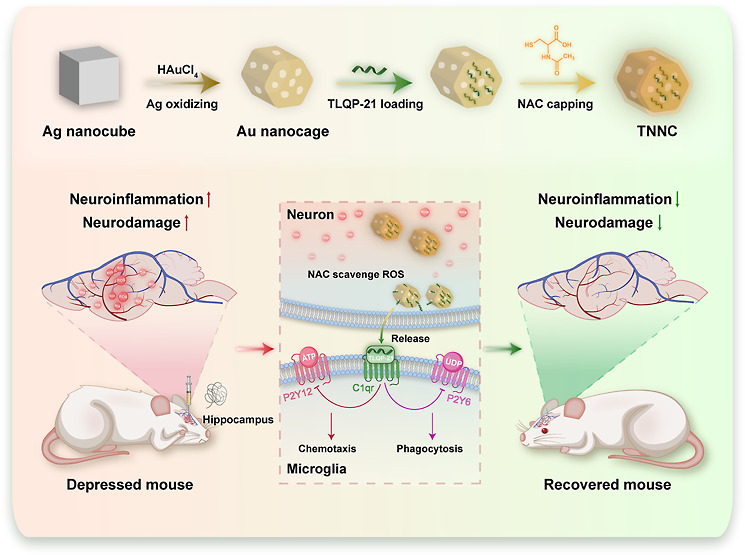

## Introduction

Major depressive disorder (MDD) is a common psychiatric disorder characterized by persistent depressed mood accompanied by deficits in daily living functioning, which severely limits psychosocial functioning, decreases the quality of life, and has become a global health crisis.^1,2^ To date, the pathogenesis of MDD remains unclear, and multiple hypotheses have been proposed.^3^ Robust evidence suggests that neurotrophic factor disorder and oxidative stress are key elements in the pathogenesis of MDD.^3^

Neurotrophic factors, particularly the brain-derived neurotrophic factor (BDNF), are vital to neuronal growth and survival, synaptic neurotransmission, and neuroplasticity in brain circuits involved in emotional and cognitive functions. The neurotrophic hypothesis of depression proposes that depression results from the decline of BDNF levels in the brain. BDNF exerts antidepressant effects by modulating downstream targets, including VGF nerve growth factor inducible (VGF), a neuropeptide precursor.^4,5^ The neuroprotection and neuro-recovery effects of multiple peptides with the *N*-terminal sequence Thr-Leu-Gln-Pro (TLQP) derived from the VGF precursor protein, such as the 21-amino acid peptide with TLQP (TLQP21) and the 62-amino acid peptide with TLQP (TLQP62), have been reported on various neurological disorder animal models, although the exact molecular mechanisms remain largely unknown.^6–8^ Among these VGF derivatives, TLQP21 is one of the most studied by virtue of its small size, endocrine and external secretion effects, antiapoptotic potential, and many other roles in physiological and pathological events,^9–11^ implying TLQP21 is a promising antidepressant therapeutic for MDD. Notably, neurotrophic factors and their derivatives suffer from oxidative damage in vivo, which suggests the importance of delivery strategy to protect TLQP21 from in vivo oxidative damage.^12^

Oxidative stress is another significant pathogenic factor in MDD, as the brain is particularly vulnerable to oxidative stress due to its elevated oxygen consumption, higher lipid content, and relatively weaker antioxidative defenses.^13^ Oxidative stress induced by excessive production and accumulation of reactive oxygen species (ROS) causes brain dysfunction, neuronal plasticity, and a decrease in frontal cortical and hippocampal volumes in MDD patients.^14–17^ Therefore, antioxidants, such as *N*-acetylcysteine (NAC), have been proposed as potential MDD therapeutics. However, the application of NAC in neurological disorders remains limited by its low bioavailability, poor accumulation, and retention of lesions in vivo.

To date, there remains a lack of efficient delivery systems targeting neurotrophic factor disorder and oxidative stress in MDD. Gold nanocages (AuNCs) with shell structures are used for controlled drug release and photothermal therapy due to multiple advantages, including but not limited to their (1) outstanding biocompatibility, (2) typical hollow structures and ultrathin porous walls to enhance drug loading and achieve controlled release, (3) easily adjusted size to optimize cell uptake and distribution, (4) flat atomic planes with excellent stability and flexibility, (5) easily controlled position of the localized surface plasmon resonance (LSPR) peak to specific wavelength in the near-infrared (NIR) window, and (6) high extinction coefficient and photothermal conversion efficiency in photothermal therapy.^18–21^ In this study, we utilized AuNCs to establish a two-pronged joint intervention strategy for MDD. We packaged TLQP21 within AuNCs and sealed holes on the surface of AuNCs by NAC with Au–S bonding (TLQP21-entrapped and NAC-capped AuNC, TNNC). AuNCs and NAC protected TLQP21 from oxidative and other potential damages in a normal state. Under hyper-oxidative stress conditions, NAC exerted its antioxidative functions to eliminate excessive ROS. The consumption of NAC further released TLQP21 from AuNCs to activate BDNF-VGF signaling, rectifying neurotrophic factor disorder in MDD. Our results suggested that TNNC significantly alleviated chronic unpredictable mild stress (CUMS)-induced depression-like behaviors in mice, reduced ROS levels, and inhibited neural damage in mouse brains. Our results further suggested that TNNC exerted its therapeutic effects on depression-like phenotypes presumably via manipulating the activation and synaptic pruning capacity of microglia. TLQP21 mediated TNNC’s regulatory roles in microglia through activating complement C1q receptor (C1qR) and complement C3a receptor 1 (C3aR1), resulting in the inhibition of its downstream factors Purinergic receptor P2Y, G-protein coupled, 12 (P2Y12), and Purinergic receptor P2Y, G-protein coupled, 6 (P2Y6). Hence, our study designed a promising nanoparticle to intervene in two distinct pathological pathways in MDD at the same time, shedding light on the development and clinical applications of nanocarriers for multiple pharmacological effects.

## Results and Discussion

### Preparation and Physicochemical Characterization of TNNC

AuNCs were prepared and coated with NAC as NAC-capped AuNCs (NNC). As shown in Figure 1a,b, the hydrodynamic diameter of NNC was 49.0 nm, and the polydispersity index was 0.138, indicating uniform size distribution. Meanwhile, a similar nanocage structure was observed via transmission electron microscopy (TEM) and scanning electron microscopy (SEM). Besides nanomorphology, the S–H stretching vibration peak in the Raman spectrum at 2547 cm^–1^ disappeared in NNC, confirming that NAC molecules were successfully anchored onto the surface of AuNCs through Au–S bonding^22^ (Figure 1c). The biosafety of AuNCs was examined in vivo by injecting AuNCs into the hippocampal tissues of wild-type (WT) mice (Figure S1). Gold metabolism analysis demonstrated gradual degradation of AuNCs in the brain from post-injection day 1 to day 7 (Figure S1a). More importantly, there were no significant differences in the numbers of TUNEL^+^ apoptotic cells and NeuN^+^ neuronal cells in the hippocampi 1, 3, or 7 days post AuNC injection, compared with controls, suggesting negligible neurotoxicity of AuNCs (Figure S1b–d).

**Figure 1:**
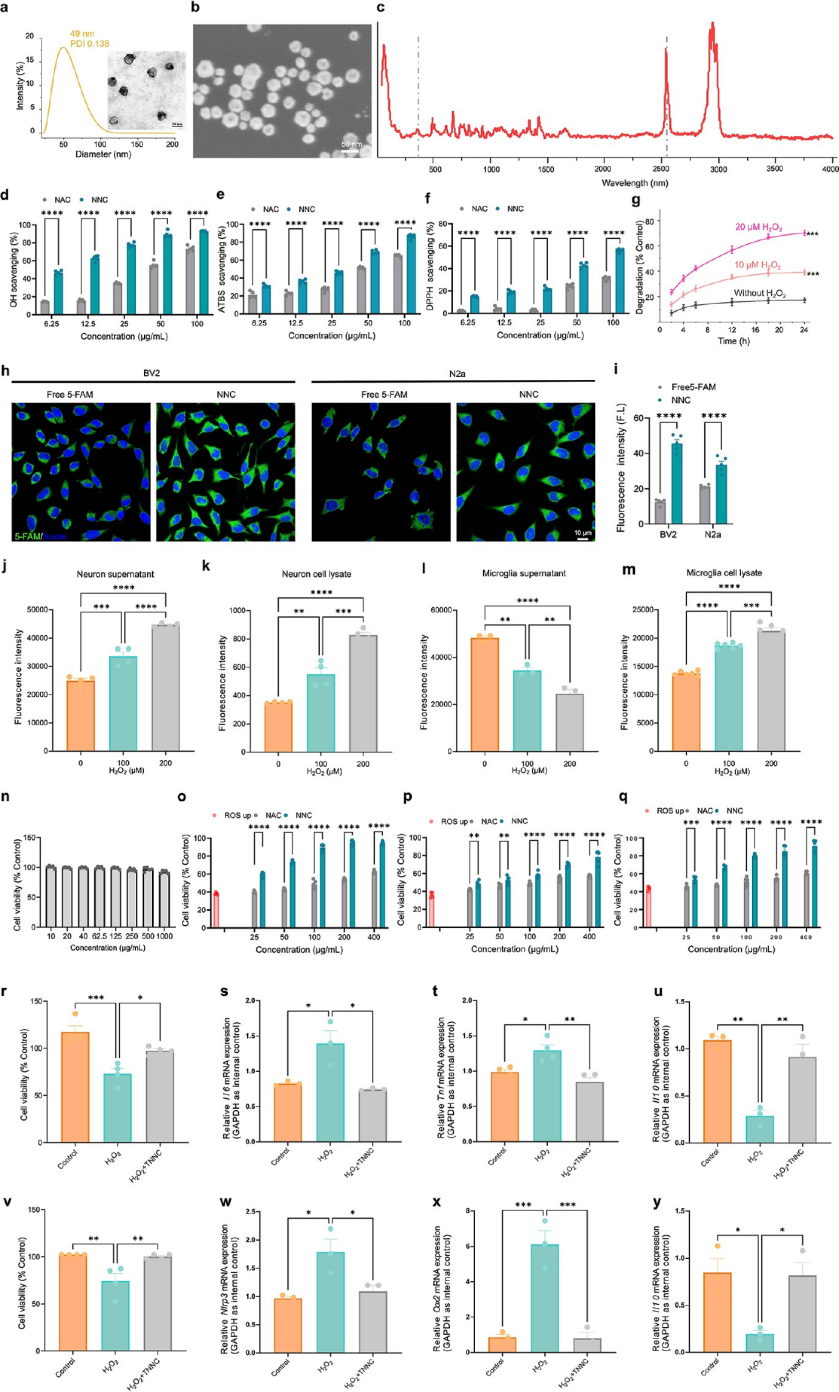
The synthesis, characterization, and function of NNC/TNNC. (a,b) The hydrate diameter (a) and microstructure (b) of NNC. Scale bar: 50 nm. (c) The Raman spectrum of NNC. (d,e) OH^•^ (*n* = 3, unpaired *t*-test) (d) and ATBS^•^ (*n* = 3, unpaired *t*-test) (e) scavenging capacity of NAC or NNC at the same NAC concentration gradient (*n* = 3, unpaired *t*-test). (f) DPPH^•^ scavenging capacity of NAC or NNC at the same NAC concentration gradient (*n* = 3, unpaired *t*-test). (g) The degradation and encapsulated probe release under an excessive oxidative stress-stimulated environment (*n* = 3, one-way ANOVA). (h) CLSM imaging of BV2 or N2a cells after 12 h of incubation with free 5-FAM or 5-FAM-labeled NNC. Scale bar: 10 μm. (i) Confocal semiquantitative analysis of intracellular fluorescence intensity (*n* = 5, two-way ANOVA). (j) Quantitative analysis of released TLQP21-FAM in cultured neuron supernatant after treatment with different concentrations of H_2_O_2_ and TNNC-FAM (*n* = 4, one-way ANOVA). (k) Quantitative analysis of uptake of TLQP21-FAM by cultured neurons after treatment with different concentrations of H_2_O_2_ and TNNC-FAM (*n* = 4, one-way ANOVA). (l) Quantitative analysis of released TLQP21-FAM in cultured microglia supernatant after treatment with different concentrations of H_2_O_2_ and TNNC-FAM (*n* = 4, one-way ANOVA). (m) Quantitative analysis of uptake of TLQP21-FAM by cultured microglia after treatment with different concentrations of H_2_O_2_ and TNNC-FAM (*n* = 4, one-way ANOVA). (n) The biocompatibility investigation of AuNC and NNC in BV2 cells (*n* = 3). (o–p) Cell viability of BV2 cells (*n* = 3, unpaired *t*-test) (o), N2a cells (*n* = 3, unpaired *t*-test) (p), or BV2–N2a coincubation system (*n* = 3, unpaired *t*-test) (q) after ROSup exposure and NAC/NNC treatment (*n* = 3, unpaired *t*-test). (r) CCK8 assay of BV2 cell viability after H_2_O_2_ exposure and TNNC treatment (*n* = 4, one-way ANOVA). (s–u) Quantification of the transcriptional levels of *Il6* (s), *Tnf* (t), and *Il10* (u) in H_2_O_2_- and TNNC-treated BV2 cells (*n* = 4, one-way ANOVA). (v) CCK8 assay of N2a cell viability after H_2_O_2_ exposure and TNNC treatment (*n* = 4, one-way ANOVA). (w–y) Quantification of the transcriptional levels of *Nlrp3* (w), *Cox2* (x), and *Il10* (y) in H_2_O_2_- and TNNC-treated N2a cells (*n* = 4, one-way ANOVA). All data are represented as means ± s.d. ****p < 0.0001, ***p < 0.001, **p < 0.01, and *p < 0.05.

Afterward, the antioxidant ability of NNC was assessed by the hydroxyl (^•^OH) and 2,2-azino-bis (3-ethylbenzothiazoline-6-sulfonic acid) (ABTS^•^) free radical scavenging capacity assay kit, and the scavenging ratio is exhibited in Figure 1d,e. Although the concentration-dependent ^•^OH scavenging could be observed in both the free NAC and NNC groups, the scavenging capacity of NNC was much higher than that of free NAC, due to the nanoshell structure enhancing catalysis. As shown in Figure 1f, a concentration-dependent and high level of nitrogen-centered radical scavenging was observed in the NNC group, which could be beneficial for treating complex excessive oxidative stress-caused diseases. The dose- and time-dependent release was observed during incubation with hydrogen peroxide (H_2_O_2_) solution (Figure 1g). The effectiveness of NNC uptake by glial and neuronal cells was verified via mouse microglia (BV2) and mouse neuroblastoma (N2a) cell lines, respectively. The bright green fluorescence signal was observed in both BV2 and N2a cells after incubation with 5-FAM-labeled NNC, which was a 3.42-fold or 1.59-fold increase compared with the control group (Figure 1h,i). “The Oxidative Stress Hypothesis for Depression” proposes that increased production of ROS and depletion of antioxidant defenses are the main reasons for the structural and functional changes in the brains of MDD patients.^23–25^ Therefore, we used H_2_O_2_ to simulate the oxidative stress environment and further explored the uptake of TLQP21 under oxidative stress. First of all, we performed flow cytometry and immunofluorescence to assess the oxidative stress levels in primary cultured neurons after treating them with different concentrations of H_2_O_2_.^26^ We found that both 100 and 200 μΜ Η_2_Ο_2_ treatments significantly elevated ROS levels of primary neurons (Figure S2a–d). Afterward, we incubated TNNC-FAM with primary cultured neurons in the oxidative stress environment and collected the supernatant and cell lysates to detect fluorescent intensity (Figure 1j,k). Different from the results of microglia (Figure 1l,m), we found that neurons under oxidative stress released more TLQP21 into the supernatant. These results suggested that in the oxidative stress environment neurons released more TLQP21 into the extracellular region compared with microglia.

### TNNC Mitigated Oxidative Stress and Inflammation In Vitro

Prior to intracellular and in vivo investigation, the biocompatibility of AuNC and NNC was studied by using the CCK-8 assay. As shown in Figure 1n, no significant change in cell viability was observed under incubation with 1 mg/mL of AuNCs. ROSup was further used to validate the ability of NNC in intracellular ROS and reactive nitrogen species (RNS) scavenging. In addition, the BV2 and N2a coincubation system was introduced for more accurate modeling of cellular distribution and antioxidant effects in the brain.^27^ As shown in Figure 1o–q, the cell viability was increased to 61.3% or 91.8% after incubation with free NAC or NNC, which could contribute to the high level of cell uptake and the antioxidant ability of intracellular NNC. Although low levels of cell survival were observed in N2a cells even under incubation with 400 μg/mL of NNC, the BV2–N2a coculture group was able to maintain high levels of cell survival. Besides, Rosup induced high ROS expression. We also used 600 μM H_2_O_2_ to mimic oxidative stress in the BV2 and N2a cells.^28^ We detected the cell viability of BV2 and N2a by CCK8 and found that the cell viability decreased significantly after H_2_O_2_ treatment and increased significantly after TNNC treatment (Figure 1r,v). Meanwhile, we also used quantitative reverse transcription-polymerase chain reaction (RT-qPCR) to detect the expression levels of pro-inflammatory factors and anti-inflammatory factors under oxidative stress. Our results revealed that H_2_O_2_ treatment significantly increased the expression levels of interleukin-6 (*Il6*) and tumor necrosis factor-alpha (*Tnf*) and decreased the expression of interleukin-10 (*Il10*) in the BV2 cells. Notably, TNNC treatment largely reversed the elevated expression of pro-inflammatory factors and the decreased expression of anti-inflammatory factors (Figure 1s–u). A Western blotting assay also showed that the protein levels of pro-inflammatory TNF-α and IL-6 were significantly decreased, and those of anti-inflammatory IL-10 were significantly increased in H_2_O_2_-exposed BV2 cells after TNNC treatment (Figure S3a–d). Similarly, in the N2a cells, TNNC treatment effectively reversed the increased expression of transcripts corresponding to the nucleotide-binding oligomerization domain, leucine-rich repeat, pyrin domain-containing 3 (*Nlrp3*), and cyclooxygenase-2 (*Cox2*) and largely reversed the H_2_O_2_-induced decrease in the expression of *Il10* transcript levels (Figure 1w,y). TNNC treatment also downregulated protein levels of NLRP3 and Cox2 and elevated IL-10 protein levels in H_2_O_2_-exposed N2a, validating RT-qPCR results (Figure S4a–d). The above results showed that TNNC treatment effectively reduced cell death under oxidative stress and the overproduction of pro-inflammatory factors in vitro.

### TNNC Mitigated Depressive Behaviors of CUMS Mice

To investigate the antidepressant effects of TNNC, we established a CUMS mouse model to mimic the clinical presentations of MDD. Mice were exposed to the CUMS protocol for 3 weeks, and behavioral tests assessing depressive-like symptoms were conducted to distinguish CUMS-resilient and susceptible mice because not all mice exposed to CUMS are susceptible to these effects. Global activities in the tail suspension test (TST) and forced swimming test (FST) were significantly decreased in the CUMS group compared to the control group (Figure S5a,c). Meanwhile, the percentage of immobility time in FST also increased in the CUMS group compared to the control group, but the immobility time in the TST showed no significant difference between the control group and the CUMS group (Figure S5b,d). To further exclude the resilient mice, which are unsusceptible to CUMS treatment, we distinguished the susceptible mice and the resilient mice via the global activities of TST. We found that mice susceptible to CUMS showed reduced global activities and prolonged immobility durations in TST compared to CUMS-resilient ones and controls (Figure S2e,f). In the FST, CUMS resulted in a significant reduction in global activities and an increase in the percentage of immobility time compared with wild type (WT) mice, but there was no significant difference between the CUMS susceptible group and the resilient group (Figure S2g,h). The above results revealed the successful establishment of the CUMS-induced MDD mouse model, and CUMS-susceptible mice (CUMS mice) were selected for the following studies. CUMS mice were then injected once with 2 μL of TNNC (1 mg/mL) via bilateral stereotactic injection. Depressive-like behavioral tests were conducted 3 days post-TNNC treatment. The administration of TNNC, but not that of NAC and NNC, significantly increased the global activities and decreased the immobility time in CUMS mice compared to CUMS controls in the TST (Figure 2a–c). Similarly, TNNC administration reduced the immobility time of CUMS mice in the FST (Figure 2d–f). In contrast, NAC or NNC treatments did not decrease the immobility time of CUMS mice in the TST (Figure 2b,c). Hence, our results suggested that TNNC alleviated depressive behaviors in CUMS mice.

**Figure 2:**
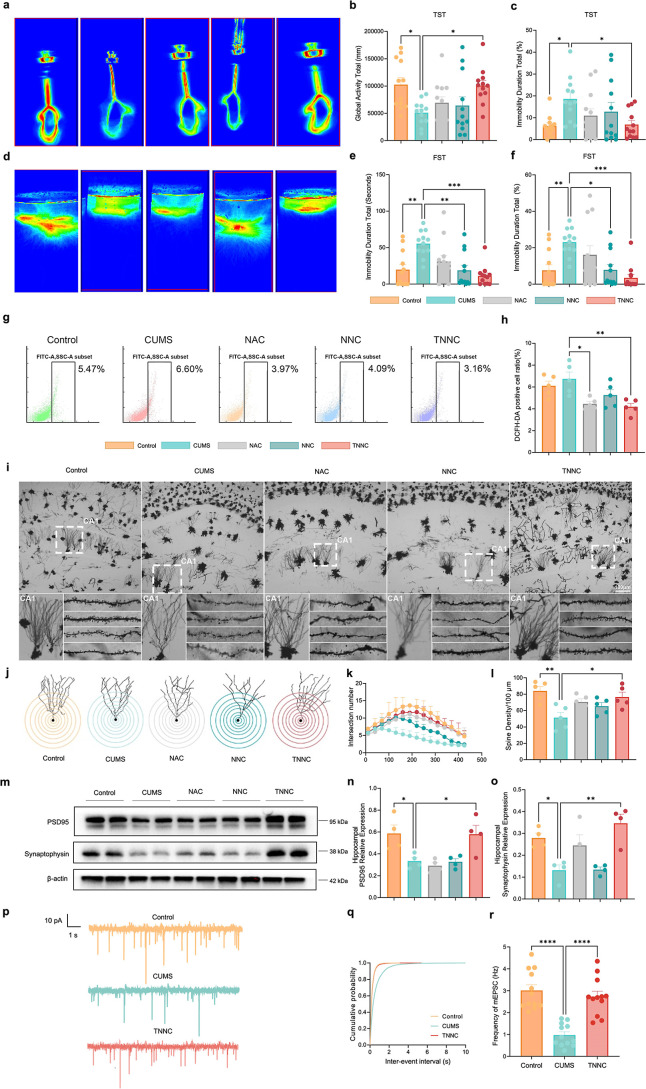
TNNC mitigated CUMS-induced depressive behaviors, ROS accumulation, and neuronal damage. (a) Representative heat map of the tail suspension experiment. (b,c) The global activities (b) and percentage of immobility condition (c) of control mice and CUMS mice treated with PBS, NAC, NNC, or TNNC in the tail suspension test (control *n* = 12, CUMS *n* = 12, NAC *n* = 12, NNC *n* = 12, TNNC *n* = 12, one-way ANOVA). (d) Representative heat map of the forced swimming experiment. (e,f) The immobility time (e) and percentage of immobility condition (f) of control mice and CUMS mice treated with PBS, NAC, NNC, or TNNC in the forced swimming test (control *n* = 12, CUMS *n* = 12, NAC *n* = 12, NNC *n* = 12, TNNC *n* = 12, one-way ANOVA). (g) Brain ROS levels of control mice and CUMS mice treated with PBS, NAC, NNC, or TNNC by flow cytometry analysis (10,000 cells per group). (h) Quantification results of flow cytometry analysis (*n* = 5, one-way ANOVA). (i) Representative images of Golgi staining for neurons and dendritic spines in the hippocampi of control mice and CUMS mice treated with PBS, NAC, NNC, or TNNC. Scale bar: 100 μm. (j) Tracing of representative hippocampal pyramidal neurons in the brains of control mice and CUMS mice treated with PBS, NAC, NNC, or TNNC. (k) Sholl analysis presenting the numbers of intersections versus the distance to the soma (*n* = 5, one-way ANOVA). (l) Quantification of spine density of dendritic spines in the hippocampi of control mice and CUMS mice treated with PBS, NAC, NNC, or TNNC (*n* = 5, one-way ANOVA). (m) Representative immunoblots for postsynaptic protein PSD95 and presynaptic protein synaptophysin using the brain lysates of control mice and CUMS mice treated with PBS, NAC, NNC, or TNNC. (n,o) Quantitative results of PSD95 (n) and synaptophysin (o) expression levels among all groups (*n* = 4, one-way ANOVA). (p) Representative postsynaptic current (mEPSC) from whole cell recordings of spontaneous excitatory activities of hippocampal neurons in control, CUMS, and TNNC groups. (q,r) Cumulative fraction plots of mEPSC interevent interval (q) and frequencies (r) (*n* = 12, one-way ANOVA). All data are represented as means ± s.d. ****p < 0.0001, ***p < 0.001, **p < 0.01, and *p < 0.05.

### TNNC Alleviated Oxidative Stress and Neuronal Damage in the Brains of CUMS Mice

To explore the mechanism of alleviation of the depressive process in CUMS mice, we examined the effect of TNNC on ROS levels in the hippocampi of CUMS mice by flow cytometry. Our results showed that both NAC and TNNC reduced ROS levels in the hippocampi of CUMS mice (Figure 2g,h). Consistent with flow cytometry results, TEM results showed that the disruption of mitochondrial membrane integrity (white arrow) and the number of vesicles decreased in presynaptic membranes of CUMS mice (yellow arrow) (Figure S6a,b). Besides, immunofluorescence staining results showed that the oxidative stress marker protein Cox2 was mainly expressed in neurons but not in microglia or astrocytes in the Dentate Gyrus (DG) region, which indicated that oxidative stress has a significant impact on hippocampal neurons (Figure S7a,b).

Oxidative stress causes brain dysfunction, neuronal plasticity, and a decrease in the volume of the frontal cortical and hippocampal tissues in patients with MDD.^14^ Therefore, the effect of TNNC on the synaptic structure of neurons in CUMS mice was next examined by Golgi staining. The results revealed impaired neuronal arborization and a reduced number of dendritic spines in the hippocampi in CUMS mice, and this damage was salvaged by TNNC as both the quantification of the CA1 pyramidal cells’ apical dendrites and the spine density of dendritic spines were increased (Figure 2i–l). Western blotting further showed significantly decreased expression levels of the presynaptic marker synaptophysin and the postsynaptic marker PSD95 in the hippocampi of TNNC-treated CUMS mice (Figure 2m–o). Based on the above findings, we then evaluated the electrophysiological properties of neurons. The impairments of synaptic plasticity, the decrease in glutamatergic synaptic density, and the synapse deficits observed in CUMS mice were also largely restored by TNNC injection (Figure 2p,r).

### Transcriptomics Analysis of Mice after TNNC Treatment

Our previous studies have examined how TNNC treatment alleviates oxidative stress to rescue neuronal injury. To further explore gene expression changes among WT mice, CUMS mice, and TNNC-treated CUMS mice, we evaluated the transcriptomic features of the hippocampi of WT and CUMS mice with or without TNNC treatment through bulk RNA sequencing analysis (RNA-seq). The principal component analysis (PCA) diagram highlights the variation in gene expression across the different groups. The first two principal components revealed a clear separation between the CUMS group and both the control and TNNC groups, suggesting distinct patterns of gene expression among these groups (Figure 3a). This was further corroborated by the hierarchical clustering heatmap in Figure 3b, which showed that the control and TNNC groups formed distinct clusters with the CUMS group. The volcano plot showed the fold change and statistical significance of gene expression differences between the CUMS mice with and without TNNC treatment. In the upregulation gene set, chemokine genes (C–C motif chemokine ligand 2 (*Ccl2*), C–C motif chemokine ligand 3 (*Ccl3*), C–C motif chemokine ligand 5 (*Ccl5*), C–C motif chemokine ligand 6 (*Ccl6*), C–C motif chemokine ligand 9 (*Ccl9*), C–X–C motif chemokine ligand 5 (*Cxcl5*), C–X–C motif chemokine ligand 6 (*Cxcl6*), C–X–C motif chemokine ligand 9 (*Cxcl9*)), complement genes (complement C1q A chain (*C1qa*), complement C1q B chain (*C1qb*), complement C1q C chain (*C1qc*)), Toll-like receptor genes (Toll-like receptor 1 (*Tlr1*), Toll-like receptor 2 (*Tlr2*), Toll-like receptor 4 (*Tlr4*)), integrin genes (integrin subunit alpha x (*Itgax*), integrin subunit beta 2 (*Itgb2*)), and disease-associated microglia (DAM) genes (triggering receptor expressed on myeloid cells 2 (*Trem2*), Lymphocyte antigen 86 (*Ly86*), TYRO protein tyrosine kinase binding protein (*Tyrobp*), C-type lectin domain family 7 member A (*Clec7a*)) were all included, which indicated TNNC treatment effectively reduced inflammatory response of CUMS mice (Figure 3c). Consistent with the volcano plot result, Gene Ontology (GO) enrichment analysis of the top 20 enriched terms revealed that inflammation-associated pathways such as the immune system process, innate immune process, response to bacterium, immune response, inflammatory response, defense to virus, cellular response to interferon-beta, neutrophil chemotaxis, positive regulation of tumor necrosis factor procession, and chemokine activity were significantly enriched, indicating that these biological processes were strongly affected by TNNC treatments (Figure 3d). Meanwhile, KEGG (Kyoto Encyclopedia of Genes and Genomes) analysis revealed that TNNC treatment mainly affected the following signaling pathways: NOD-like receptor signaling pathway, rheumatoid arthritis, leishmaniasis, cytokine–cytokine receptor interaction, phagosome, influenza A, *Staphylococcus aureus* infection, pertussis, Epstein–Barr virus infection, and viral protein interaction with cytokine and cytokine receptor (Figure 3e). Gene Set Enrichment Analysis (GSEA) was used to evaluate the enrichment of specific gene sets related to immune and disease pathways. According to the KEGG analysis result, the NOD-like receptor signaling pathway (mmu04621) (*p* < 0.001, FDR < 0.001) and phagosome pathway (mmu04145) (*p* < 0.001, FDR < 0.001) were significantly enriched in the CUMS group, indicating that TNNC treatment alleviated inflammation and phagocytosis (Figure 3f,g). Furthermore, the heatmap shown in Figure 3h,i provided further insight into the expression pattern of genes involved in the NOD-like receptor signaling pathway and the phagosome pathway, respectively. In the NOD-like receptor signaling pathway, immune-related genes such as *Ccl2*, apoptosis regulatory kinase *Caspase4* (*Casp4*), and pro-inflammatory cytokine *interleukin-1 beta* (*Il1b*) were upregulated in the CUMS group, confirming the activation of this immune pathway (Figure 3h). Similarly, phagocytosis-related genes such as complement receptor *C1ra* and C-type lectin domain family 7 member A (*Clec7a*) were also upregulated in the CUMS group, suggesting that TNNC treatment alleviated the excessive phagocytosis of immune cells (Figure 3i). To validate our findings, we examined the expression of *Ccl2*, *Casp4*, *Clec7a*, *Il1b*, and *C1ra*, the 5 genes mentioned above that are strongly associated with neuroinflammation and phagocytosis, via RT-qPCR (Figure S8a–e). The results revealed that TNNC treatment significantly decreased the level of CUMS-induced excessive expression of the aforementioned genes, confirming the RNA-seq results. These findings suggested that TNNC played a significant role in modulating immune responses, particularly through the activation of immune pathways involved in pathogen recognition and phagocytosis.

**Figure 3:**
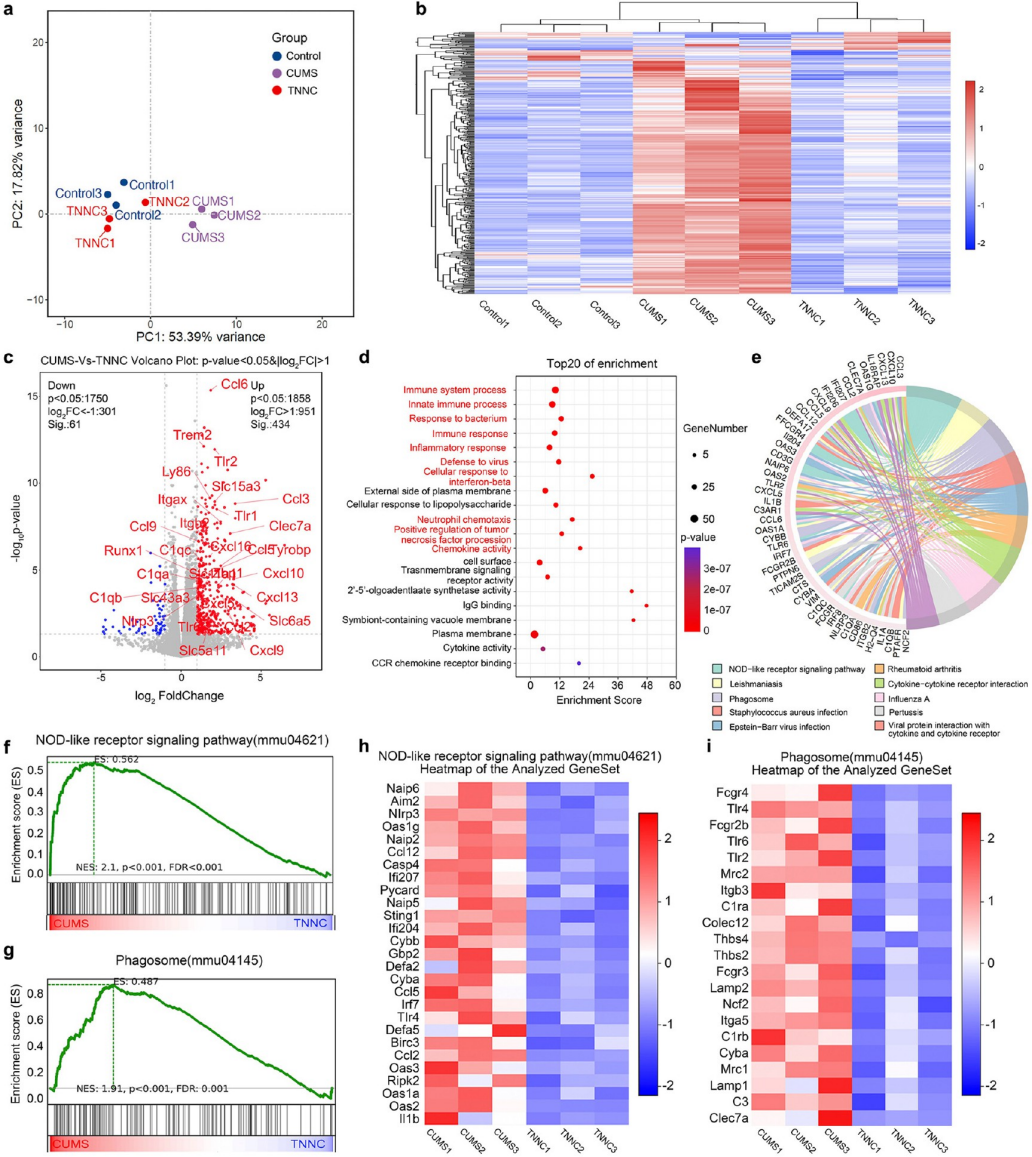
Transcriptome sequencing of TNNC-treated CUMS mouse hippocampi. (a) Principal component analysis (PCA) plot of control, CUMS, and TNNC in RNA-seq (b) DEG heatmap among the control, CUMS, and TNNC treatment groups. (c) A volcano plot of gene expression changes from CUMS-treated WT mice compared with TNNC-treated CUMS mice. (d) GO analysis identified the top 20 significantly enriched GO terms of DEGs. (e) KEGG analysis of the correlation between the KEGG enrichment pathway and DEGs. (f) GSEA enrichment plot of the NOD-like receptor signaling pathway. (g) GSEA enrichment plot of the phagosome pathway. (h) The heatmap of the GSEA-enriched NOD-like receptor signaling pathway between the CUMS and TNNC groups. (i) The heatmap of the GSEA-enriched phagosome pathway between the CUMS and TNNC groups.

### TNNC Inhibited Depression-Associated Activation, Inflammatory Responses, and Excessive Synaptic Pruning of Microglia

Since microglia are the resident macrophages that play a central role in mediating immune responses in the central nervous system,^10^ we then examined the effects of TNNC treatment on microglial activation in the brains of CUMS mice. Immunofluorescence staining results showed a significant reduction of ionized calcium-binding adapter molecule 1^+^ (Iba1^+^) microglia and glial fibrillary acidic protein^+^ (GFAP^+^) astrocytes in the hippocampi after TNNC treatment versus CUMS mice, suggesting that TNNC inhibited CUMS-induced glia activation (Figure S9a–d). TNNC also reversed CUMS-induced morphological changes of microglia, ascertained by the increased branch numbers and length of microglia and reduced microglial body volumes in the hippocampi of CUMS mice after TNNC administration (Figure 4a–e). In addition, Western blotting results showed that the protein levels of pro-inflammatory factors, including NLRP3, Cox2, and nuclear factor-κ-gene binding (NF-κB), were reduced in the hippocampi of CUMS mice after TNNC treatment but not after NAC or NNC administration (Figure 4f–i). We also found that pro-inflammatory factors TNF-α and IL-1β significantly decreased in CUMS mice after TNNC administration via an ELISA assay (Figure 4j,k).

**Figure 4:**
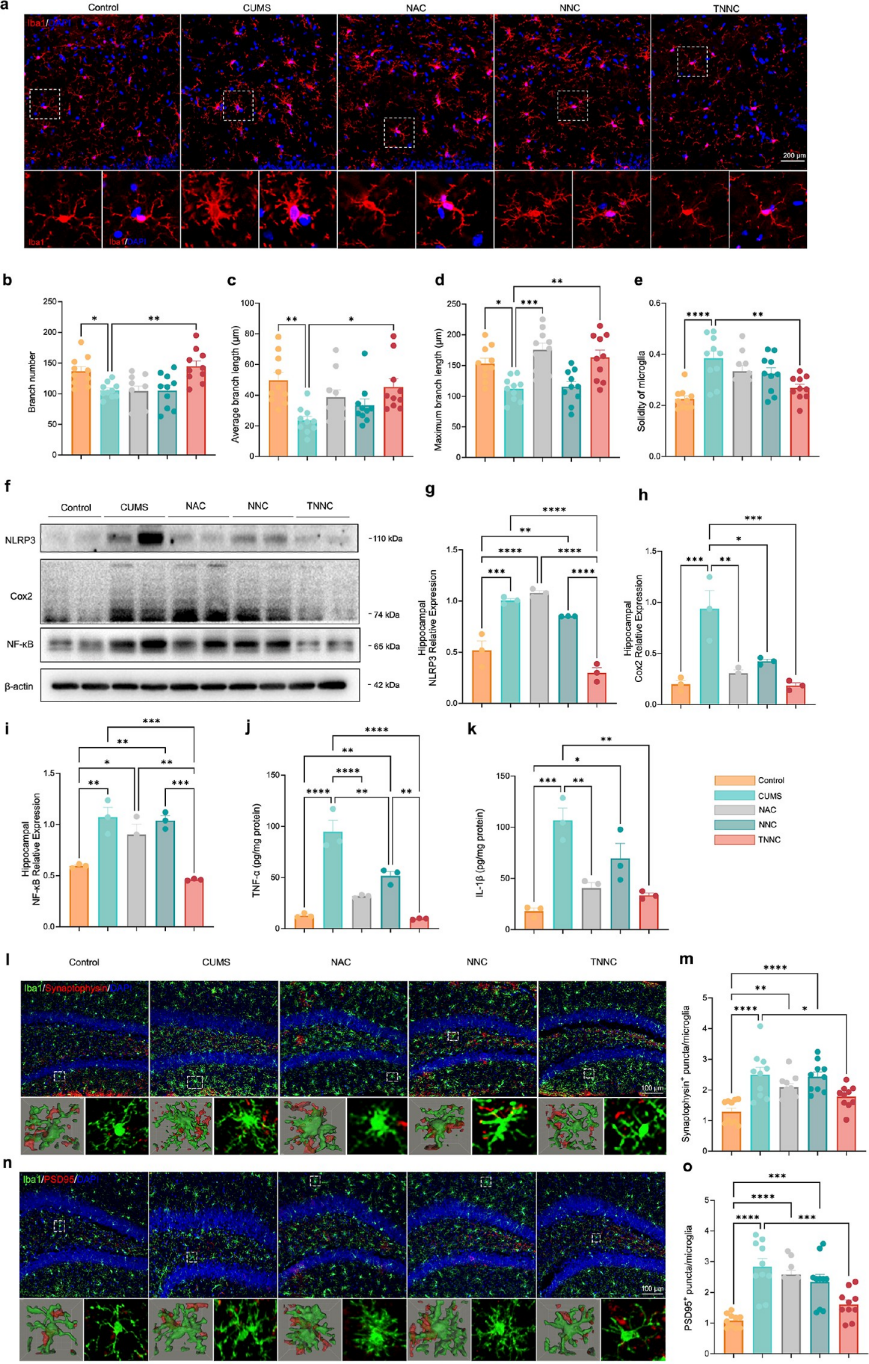
TNNC inhibited CUMS-induced inflammation and excessive synaptic pruning of microglia. (a) Representative confocal microscopic images and their zoomed pictures (white frame) demonstrating cellular morphologies for Iba1^+^ microglia in CA1 regions of hippocampi. Scale bar: 200 μm. (b–e) Quantitative results of the branch number (b), average branch length (c), maximum branch length (d), and solidity of microglia (e) for individual microglia (*n* = 10, one-way ANOVA). (f) The levels of inflammation-related genes NLRP3, Cox2, and NF-κB in protein lysates were determined by Western blotting. (g–i) Quantitative results of NLRP3 (g), Cox2 (h), and NF-κB (i) expression (*n* = 3, one-way ANOVA). (j–k) ELISA results showed the expression levels of inflammatory cytokines TNF-α (j) and IL-1β (k) in brain tissue of each group (*n* = 3, one-way ANOVA). (l) Representative confocal microscopic images of immunostainings for Iba1 and synaptophysin in DG regions. Magnification and 3D reconstructed images were placed below each panel (white frame). Scale bar: 100 μm. (m) Quantification of synaptophysin^+^ puncta numbers in Iba1^+^ cells (*n* = 10, one-way ANOVA). (n) Representative confocal microscopic images of immunostainings for Iba1 and PSD95 in DG regions. Magnification and 3D reconstructed images were placed below each panel (white frame). Scale bar: 100 μm. (o) Quantification of PSD95^+^ puncta numbers in Iba1^+^ cells (*n* = 10, one-way ANOVA). All data are represented as means ± s.d. ****p < 0.0001, ***p < 0.001, **p < 0.01, and *p < 0.05.

We next investigated the effects of TNNC on microglia-mediated synaptic pruning, which plays an important role in synaptic loss and cortical dysfunction in depression. 3D reconstruction of immunofluorescence staining results revealed increased colocalization of Iba1^+^ microglia with presynaptic protein synaptophysin (Figure 4l) and postsynaptic protein PSD95 (Figure 4n) in the hippocampi of CUMS mice, indicating that CUMS promoted microglia to phagocytose synaptic structures. Moreover, TNNC injection significantly reduced the number of synaptophysin^+^ and PSD95^+^ synaptic structures in hippocampal microglia of CUMS mice (Figure 4m,o). The data presented above suggested that TNNC inhibited microglial overactivation and excessive phagocytosis of synaptic structures, which mitigated CUMS-induced synaptic loss and neuronal dysfunction.

### TNNC Inhibited Corticosterone (CORT)-Induced Inflammatory Responses, Migration, and Phagocytosis of Microglia

To confirm our in vivo observations, we then established an in vitro model of MDD microglial activation through a one-week exposure of corticosterone (CORT) (10 nM) (Figure 5a). After CORT exposure, the ROS levels of primary microglia were significantly increased, mimicking the increase of ROS levels in the brains of CUMS mice revealed by in vivo observations. TNNC treatment significantly reduced ROS levels, matching our in vivo observations (Figure 5b,c).

**Figure 5:**
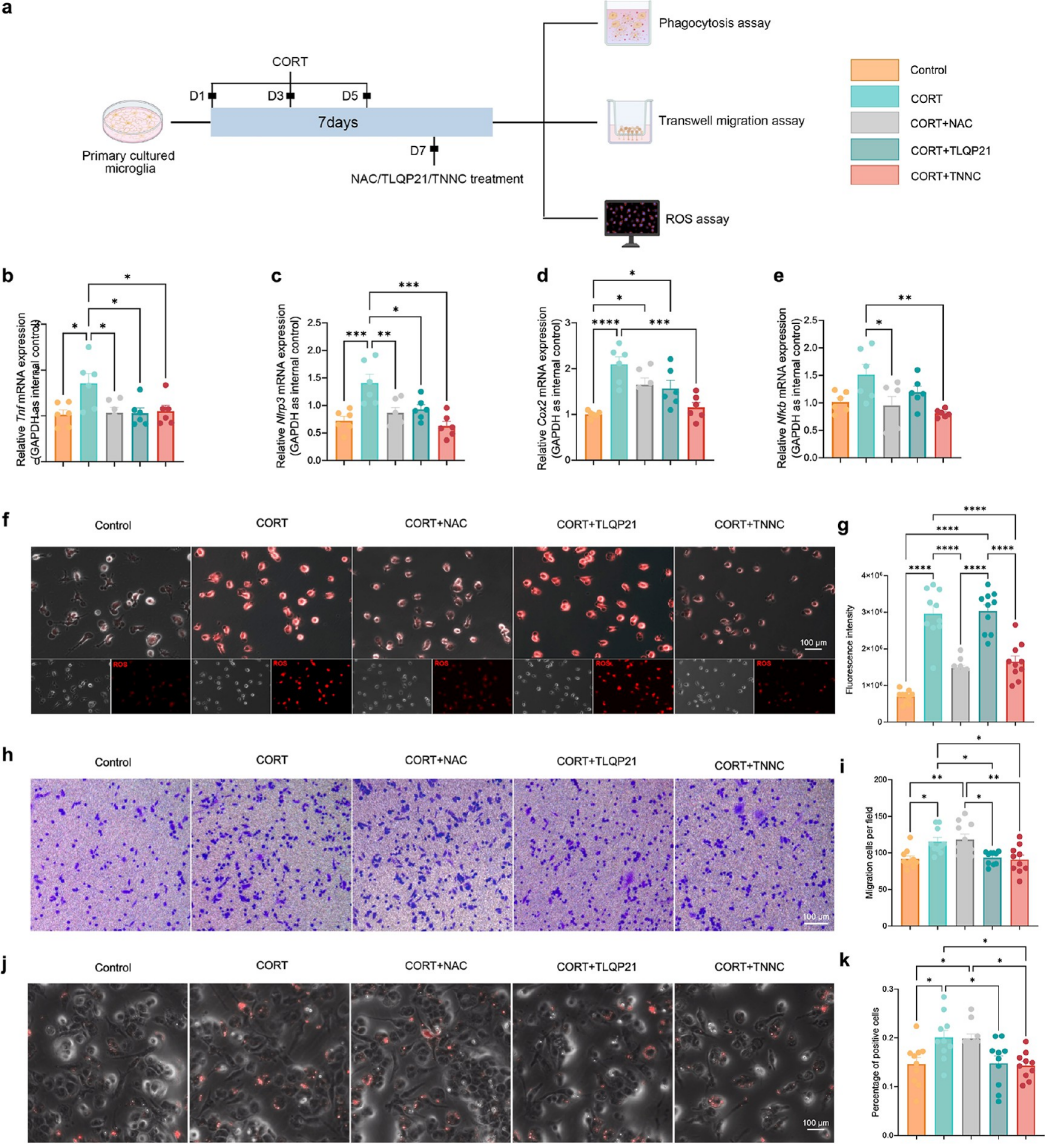
TLQP21 mediated the regulatory effects of TNNC on microglial inflammatory responses, ROS accumulation, phagocytosis, and migration. (a) Schematic diagram of experimental design. (b–e) Quantification of the transcriptional levels of *Tnf* (b), *Nlrp3* (c), *Cox2* (d), and *Nfkb* (e) in the hippocampi of each group (*n* = 6, one-way ANOVA). (f) Representative images of ROS signals of primary microglia in each group. Scale bar: 100 μm. (g) Quantification of fluorescence intensity in each group (*n* = 10, one-way ANOVA). (h) Representative images of migrated cells of each group. Scale bar: 100 μm. (i) Quantification of migrated cell numbers of each group under the chamber membrane (*n* = 10, one-way ANOVA). (j) Representative images of microglia containing latex beads in the phagocytosis assay. Scale bar: 100 μm. (k) Quantification of the percentage of microglia containing beads in different groups: in the presence of 300 μM CORT (+), phagocytosis was stimulated under control conditions, an effect that was prevented by treatment with TLQP21 and TNNC (100 nM) (*n* = 10, one-way ANOVA). All data are represented as means ± s.d. ****p < 0.0001, ***p < 0.001, **p < 0.01, and *p < 0.05. Panel (a) was created with Biorender.com.

Since TLQP21 has been demonstrated to affect microglial functions such as the secretion of pro-inflammatory molecules, phagocytic activity, and migration,^10,29,30^ we next determined the effects of TNNC and TLQP21 on microglia inflammatory responses, phagocytosis, and migration. RT-qPCR results demonstrated that TNNC treatment inhibited the inflammatory responses of primary microglia caused by CORT, ascertained by the significant reduction of expression levels of transcripts corresponding to *Tnf*, *Nlrp3*, *Cox2*, and *Nfκb* (Figure 5d–g). Microglia phagocytosis assay results also suggested that CORT stimulated primary microglia to phagocytose more fluorescent latex beads, which was abrogated by either TNNC or TLQP21 (Figure 5h,i). The Transwell assay showed that primary microglia migration was enhanced in the presence of CORT, and either TNNC or TLQP21 almost erased the effects of CORT on microglia migration (Figure 5j,k). These results revealed that TNNC or TLQP21 alone successfully alleviated microglial dysfunctions, including inflammatory responses, excessive phagocytosis, and hyper-migration under the pathological conditions of MDD.

### TNNC Regulated Microglia Activity via the C1qR-Purinergic Receptor Pathway

Next, we investigated the molecular mechanisms of the TLQP21-dependent modulation of microglia activity. Being a derivative of VGF, TLQP21 has been reported to target C1qR and C3aR1.^31^ To determine the key downstream receptors of TLQP21, we first manipulated the functions of C1qR and its downstream P2Y purinergic receptors. C1qR is a highly conserved multifunctional protein mainly present in the mitochondrial matrix.^32^ Moreover, C1qR is able to translocate from the cytoplasm to the cell membrane (lipid rafts), resulting in multiple pathological processes (e.g., metastasis of cancer cells).^33^ In microglia, C1qR has been reported to mediate chemotaxis.^34^ In brain damage, C1qR inhibited microglial migration and phagocytosis by impairing P2Y6- and P2Y12-mediated purinergic signaling, respectively (Figure 6a). We treated CORT-preincubated microglia with TLQP21 (100 nM), C1QBP polyclonal antibody (3 μg/mL) that binds to C1qR on the microglia membrane surface, and P2Y6 antagonist MRS2578 (10 μM) for 30 min to determine the roles of C1qR and P2Y6 in microglial phagocytosis. Consistent with our previous finding, TLQP21 decreased the phagocytic activity of microglia induced by CORT. However, this effect of TLQP21 was reversed by inhibiting the activity of C1qR. Moreover, MRS2578 suppressed microglial phagocytosis when C1qR was blocked, suggesting the C1qR-P2Y6 axis as a key downstream signaling pathway of TLQP21-mediated suppression of microglial hyper-phagocytosis. Similar results were obtained as that C1QBP polyclonal antibody erased the inhibitory roles of TNNC in microglial hyper-phagocytosis, which was further abrogated by MRS2578 treatment (Figure 6b–e). In addition, we also tested whether TLQP21 inhibited microglial migration via the C1qR-P2Y12 axis using the C1QBP polyclonal antibody (3 μg/mL) and P2Y12 inhibitor MRS2395 (1 μM). Transwell assay results suggested that blocking C1qR completely removed the negative effects of TLQP21 on microglial migration, and the inhibition of P2Y12 by MRS2395 reduced CORT-induced hyper-migration of microglia in the presence of the C1QBP polyclonal antibody. Similar phenomena were observed as TLQP21 was replaced with TNNC, confirming the C1qR-P2Y12 axis as the downstream effector of TLQP21 in the regulation of microglial migration (Figure 6f–i). In addition, the expression of inflammatory-related genes was analyzed by RT-qPCR. As shown in Figure 6j–m, both TLQP21 and TNNC suppressed the overproduction of inflammatory factors induced by CORT, such as *Tnf*, *Nlrp3*, *Cox2*, and *Nf*κ*b*, while the C1qR inhibitor did not reverse the treatment effects of TLQP21 and TNNC, indicating that TLQP21 suppressed microglia activity, but not inflammatory responses, via the C1qR pathway. To further evaluate the potential of the C1qR pathway as a therapeutic target of hyperactivated migration and phagocytosis of microglia, we treated CORT-exposed microglia with either MRS2395 or MRS2578 (Figure S10). Our results showed that MRS2395 significantly inhibited microglial migration and that MRS2578 significantly repressed microglial phagocytic activity, without affecting the inflammatory responses of microglia, further confirming the roles of the C1qR pathway in controlling microglial migration and phagocytosis (Figure S10a–h).

**Figure 6:**
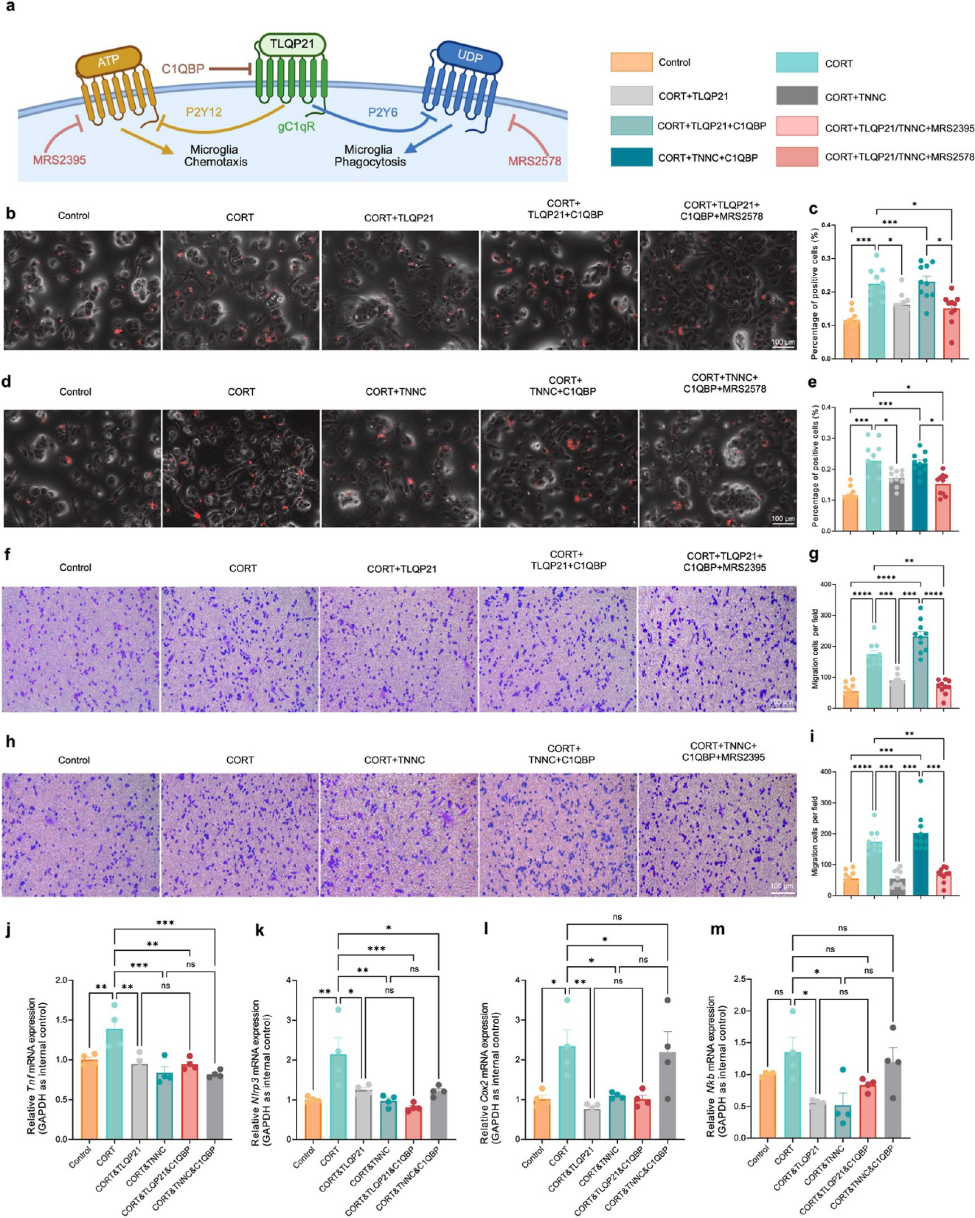
TLQP21 regulated microglial phagocytosis and migration capacities via targeting C1qR. (a) Schematic diagram of experimental design. (b) Representative images of microglia containing latex beads in the phagocytosis assay. Scale bar: 100 μm. (c) Quantification of percentages of microglia containing beads in different groups (*n* = 10, one-way ANOVA). (d) Representative images of microglia containing latex beads in the phagocytosis assay. Scale bar: 100 μm. (e) Quantification of percentages of microglia containing beads in different groups (*n* = 10, one-way ANOVA). (f) Representative images of migrated microglia treated with CORT, TLQP21, mAb C1QBP, and P2Y12 inhibitor MRS2395. Scale bar: 200 μm. (g) Quantification of migrated cells of each group under the chamber membrane (*n* = 10, one-way ANOVA). (h) Representative images of migrated microglia treated with CORT, TNNC, mAb C1QBP, and P2Y12 inhibitor MRS2395. Scale bar: 200 μm. (i) Quantification of migrated cells of each group under chamber membrane (*n* = 10, one-way ANOVA). (j–m) Quantification of the transcriptional levels of *Tnf* (j), *Nlrp3* (k), *Cox2* (l), and *Nfkb* (m) in the microglia treated with CORT, CORT&TLQP21, CORT&TNNC, CORT&TLQP21&mAb C1QBP, and CORT&TNNC&mAb C1QBP (*n* = 4, one-way ANOVA). All data are represented as means ± s.d. ****p < 0.0001, ***p < 0.001, **p < 0.01, and *p < 0.05. Panel (a) was created with Biorender.com.

### TNNC Mitigated Microglia Inflammation via the C3aR1 Receptor Pathway

Besides C1qR, we also examined the involvement of another receptor, C3aR1, in the TLQP21-mediated regulation of microglial activities. Being a 7-transmembrane G-protein-coupled receptor (GPCR), C3aR1 is predominantly expressed in macrophages, microglia, and other immune cells.^35–37^ In Alzheimer’s disease, C3aR1 is a key player in cellular metabolic regulation to control microglial response to Aβ and tau pathologies.^38,39^ Moreover, C3aR1 has been reported as the central receptor to drive microglia-mediated synaptic elimination and white matter damage after hypoperfusion^36^ and fracture surgery.^40^ At first, we pretreated microglia with CORT (10 nM, three times a week) to activate microglia. Subsequently, we treated activated microglia with TLQP21 (100 nM), TNNC (100 nM), and C3aR1 antagonist SB290157 (1 μM) that blocked C3a-induced C3aR1 internalization in a concentration-dependent manner for 3 h.^31^ Our results showed that TLQP21 reversed hypermigration (Figure 7a,b) and hyperphagocytosis (Figure 7c,d) in CORT-induced overactivated microglia with or without SB290157. Similarly, we found TNNC rescued microglial hyper-migration (Figure 7e,f) and hyperphagocytosis (Figure 7g,h), but blockage of C3aR1 antagonist SB290157 showed no effects on the migration and phagocytic capacities of microglia. Next, we performed an RT-qPCR assay to detect the expression of pro-inflammatory factors such as *Tnf*, *Nlrp3*, *Cox2*, and *Nf*κ*b* after inhibiting the receptor of C3aR1 by SB290157 in the CORT-induced primary microglia. RT-qPCR analysis revealed that TLQP21 and TNNC treatment reduced the levels of *Tnf*, *Cox2*, and *Nf*κ*b* transcripts, and the inhibitory effects of TLQP21 and TNNC on the expression of pro-inflammatory factors were reversed by C3aR1 antagonist SB290157 (Figure 7i–l), which indicated that TLQP21 alleviated microglia inflammation via the C3aR1 pathway under the pathological conditions of MDD. To further validate the functions of C3aR1 and potential C1qR-C3aR interaction in inflammatory responses of microglia, we treated CORT-exposed primary microglia with either C1QBP or t SB290157. The treatment of SB290157, but not C1QBP, significantly enhanced the levels of *Tnf*, *Cox2*, *Nlrp3*, and *Nf*κ*b* transcripts in CORT-exposed primary microglia (Figure S12a–h). These results suggested that C3aR played an important role in mediating microglial inflammatory responses without interacting with the C1qR-related pathways. Notably, we are not the first group to report distinct roles of C1qR and C3aR1 in different cellular processes of microglia under the regulation of TLQP21. Elmadany et al. have observed that TLQP21 affects purinergic signaling of microglia through the C1qR-dependent pathway and stimulates microglial phagocytic activity through C3aR1 in vitro,^10^ indicating complicated downstream targets and signaling pathways of TLQP21 in the context of neurological disorders and neuroinflammation.

**Figure 7:**
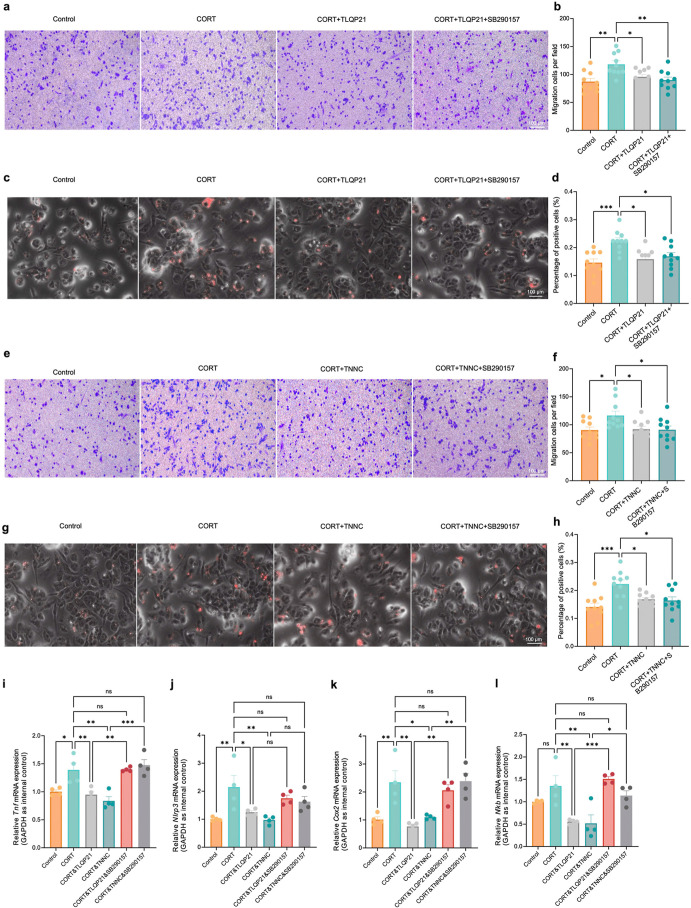
TLQP21 regulated microglial inflammatory responses via targeting C3aR1. (a) Representative images of migrated cells of microglia treated with CORT, TLQP21, and C3a receptor antagonist SB290157. Scale bar: 100 μm. (b) Quantification of migration cells of each group under the chamber membrane. A 1 mM concentration of ATP is used as a chemoattractant. We make pairwise comparisons between each group (*n* = 10, one-way ANOVA). (c) Phagocytosis assay in situ. Representative image of microglia treated with CORT, TLQP21, and C3aR1 antagonist SB290157 after 60 min incubation with latex beads. Scale bar: 100 μm. (d) Quantification of the percentage of microglia containing beads in different groups: we make pairwise comparisons between each group (*n* = 10, one-way ANOVA). (e) Representative images of migrated cells of microglia treated with CORT, TNNC, and C3aR1 antagonist SB290157. Scale bar: 100 μm. (f) Quantification of migration cells of each group under the chamber membrane. A 1 mM concentration of ATP is used as a chemoattractant. We make pairwise comparisons between each group (*n* = 10, one-way ANOVA). (g) Phagocytosis assay in situ. Representative image of microglia treated with CORT, TNNC, and C3aR1 antagonist SB290157 after 60 min incubation with latex beads. Scale bar: 100 μm. (h) Quantification of the percentage of microglia containing beads in different groups, we make pairwise comparisons between each group (*n* = 10, one-way ANOVA). (i–l) Quantification of the transcriptional levels of *Tnf* (i), *Nlrp3* (j), *Cox2* (k), and *Nfkb* (l) in the microglia treated with CORT, CORT&TLQP21, CORT&TNNC, CORT&TLQP21&C3aR1 antagonist SB290157, and CORT&TNNC&C3aR1 antagonist SB290157 (*n* = 4, one-way ANOVA). All data are represented as means ± s.d. ***p < 0.001, **p < 0.01, and *p < 0.05. ns denotes non-significance.

## Conclusions

In summary, we reported a combinatorial design approach to synthesize AuNC-based nanoparticles as potential drugs for MDD. TLQP21 was packaged within AuNCs, and NAC was used to seal holes on the surfaces of AuNCs. In oxidative stress conditions, NAC in TNNC was consumed to lower ROS levels, leading to the release of neuroprotective TLQP21. TNNC administration alleviated MDD-like behaviors of CUMS mice and inhibited ROS accumulation, neuroinflammation, and neuronal damage in the brains. We further found that TLQP21 exerted neuroprotective roles through targeting C1qR, therefore suppressing the synaptic pruning and inflammatory responses of microglia. These findings highlight the potential for developing an all-in-one approach to synthesize multifunctional and multitarget nanoparticles as therapeutics for MDD and other diseases with complicated pathogenesis.

## Experimental Section

### Fabrication of NAC-Capping Au Nanocages

A silver nanocube with a size of 50 nm was synthesized referring to the previous report method with minor modification (Toward the Synthesis of Sub-15 nm Ag Nanocubes with Sharp Corners and Edges: The Roles of Heterogeneous Nucleation and Surface Capping). Ethylene glycol was heated to 150 °C under 300 rpm of magnetic stirring for 30 min, and 600 μL of NaSH (3 mM) dissolved in ethylene glycol was rapidly added, and continuous stirring was carried out for a homogeneous solution. After reacting for 5 min, 1.25 mL of polyvinylpyrrolidone (PVP) dissolved in ethylene glycol (20 mg/mL), 0.5 mL of HCl solution (3 mM), and 0.4 mL of CF_3_COOAg (282 mM) were added sequentially under the conditions of airtight keeping to obtain a brown suspension. All solutions were cooled to room temperature (RT) by an ice-water bath and then washed twice with acetone and double-distilled water (ddH_2_O) by centrifugation at 12,000 rpm for 20 min, respectively. Then, the galvanic replacement reaction was further utilized for the preparation of Au nanocages.

Prior to AgNC suspension (100 μL) being added, 6 mL of PVP solution (1 mg/mL) was heated to boiling. HAuCl_4_ solution (0.1 mM) was then injected through a microinjection syringe with a flow rate of 0.3 mL/min until the mixture solution changed to a purple color. After the mixture was cooled to RT, excess NaCl solution was added to remove free Ag^+^. The precipitates were discarded, and the supernatant was collected and rinsed with a mixture of ddH_2_O and ethanol 3 times. NAC was dissolved to prepare NAC solution at a concentration of 1 mg/mL and then, drop by drop, added into the as-prepared AuNC suspension by peristaltic pumps at a flow rate of 1 mL/min. After 1 h of incubation, NNC was collected by centrifugation and washed with a mixture of ddH_2_O and ethanol 3 times. The AuNC and NNC suspensions were dispersed in ddH_2_O with an ultrasonic dispersion and stored at 4 °C for further experiments.

### Preparation of Fluorescence-Labeled TLQP-21

The condensation reaction between the carboxyl group of 5-carboxyfluorescein (5-FAM) and the amino group of TLQP-21 (sequence: TLQPPASSRRRHFHHALPPAR) was utilized to synthesize green fluorescence-labeled TLQP-21. In detail, 20 mg of TLQP-21 was dissolved in 2 mL of 0.01 M PBS buffer (pH 7.4), and 5 mg of 5-FAM NHS was added under 500 rpm magnetic stirring. After 4 h of reaction, the solution was dialysis purified with ddH_2_O at MWCO of 1000. Finally, the purified yellowish 5-FAM-labeled TLQP-21 (designed as 5-FAM/TLQP-21) powder was obtained by lyophilization for subsequent experiments. In addition, the fluorescence spectrum of 5-FAM/TLQP-21 was measured to ensure successful fluorescent labeling, and free TLQP-21 was set as the control group.

### Fabrication of TLQP-21-Loaded Au Nanocages

AuNCs were suspended in a 0.01 M PBS buffer (pH 7.4). A 2 mg portion of TLQP-21 was added to AuNC suspension and continuously stirred for 4 h (pH 9.0). The NAC solution was slowly dripped by a peristaltic pump at a flow rate of 1 mL/min for another 6 h of stirring at 37 °C. TNNC and NNC were purified and washed with PBS by centrifugation. The loading efficiency of TLQP-21 was determined by the fluorescence intensity of unpackaged 5-FAM-labeled TLQP-21, and NAC load capacity was assessed using the HPLC (high-performance liquid chromatography) method at 214 nm with 1.0 mL/min of flow rate and 30 °C of column temperature.

### Nanocage Characterization

Dynamic light scattering (DLS): Nano-ZS 90 Nanosizer (Malvern Instruments Ltd., Worcestershire, UK) was used to measure the hydrodynamic diameter (*D*_h_), size distribution, and zeta potential of nanocages at RT. The *D*_h_ was calculated from the computed diffusion coefficients using the Stokes–Einstein equation. Each reported measurement was conducted three times.

Transmission electron microscopy (TEM): Hippocampi specimens were immediately placed in 2.5% glutaraldehyde in 0.1 M cacodylate buffer, sectioned to ∼1 mm^2^, and incubated in the same glutaraldehyde solution for 12 h at RT. Samples were postfixed in 1% osmium tetroxide for 1.5 h and then dehydrated in increasing concentrations of alcohol, immersed in propylene oxide, and embedded in Araldite 502 resin at 60 °C. Ultrathin sections were placed on grids and stained with uranyl acetate and lead citrate. TEM images were taken with a JEOL JEM-2100F instrument at 200 kV equipped with a Gatan 894 Ultrascan 1 k CCD camera.

Scanning electron microscopy (SEM): The morphologies of the nanocages were characterized using a Zeiss Sigma 300 VP instrument. Images were recorded in secondary electron mode at 2 kV.

Fluorescence spectroscopy: Fluorescence emission spectra of fluorescence labeling were measured on a Fluorescence Spectrophotometer F-4700 (Hitachi, Ltd., Japan). The excitation and emission slit widths were both set to 5 nm. Fluorescence emission spectra were recorded at a fixed time interval.

UV–vis spectroscopy: UV–vis absorbance spectra were measured by a UV–vis spectrophotometer (INESA, China) at 25 °C (wavelength range: 200–900 nm, scan speed: middle).

Raman spectrum: A total of 10 accumulations were taken for each sample at a laser power of 80 mW with an exposure time of 60 s for each accumulation, and each sample was scanned three times at the 200–4000 cm^–1^ spectral resolution frequency. In addition, phenylalanine ((1003 ± 1) cm^–1^) was used as the normalization factor, and Peakfit version 4.12 software was used for fitting analysis.

Stability and responsiveness investigation: Nile red was selected as a fluorescence probe and loaded into NNC and then incubated with a biologically stimulated redox solution, including 10 or 20 μM H_2_O_2_. And the releasing ratio of encapsulated Nile red was detected and calculated by a fluorescence spectrophotometer during different incubation periods. In addition, NNC incubated without redox biological stimulated solution was set as the control group.

### Antioxidant Ability Investigation

The capacity of Au nanocages in various free radical scavenging was examined with the corresponding kit according to the supplier’s guidance, including the Hydroxyl Free Radical Scavenging Capacity Assay Kit, ABTS Free Radical Scavenging Capacity Assay Kit, and DPPH Free Radical Scavenging Capacity Assay Kit (Reactive Nitrogen Species, RNS). Using ATBS^•^ scavenging detection as an example for illustration, NAC and NNC solutions (50 μL) were prepared with the same NAC concentration gradient and then incubated with ABTS^+•^ work solution at RT. After 6 min of standing in a dark condition, the special UV absorption value change at 405 nm was detected to calculate the scavenging efficiency. The ABTS^+•^ work solution was treated with 50 μL of ddH_2_O or vitamin C as negative or positive controls, respectively.

### Biocompatibility Investigation

The mouse microglia (BV2) cell line was chosen for investigating the biocompatibility of various formulations by the CCK-8 assay. BV2 cells were applied from Procell and cultured with DMEM medium with 10% FBS and 1% penicillin–streptomycin in a cell incubator at 5% CO_2_. BV2 cells were digested during the logarithmic growth phase and cultured in 96-well plates at a density of 1 × 10^5^ cells/well. After all cells adhered to plates, TNNC and NNC solutions were added for 24 h of incubation, and CCK-8 solution was then added for another 1 h of incubation. The content of water-soluble formazan had a positive correlation with cell viability, which could be detected by its absorption intensity at 450 nm with a multimode reader.

### Cell Uptake Level

BV2 cells and mouse neuroblastoma (N2a) cells were applied by Sunncell with ATCC standard and cultured by cell crawls in 6-well plates. 5-FAM-labeled TLQP-21 was used to prepare 5-FAM-labeled NNC and then added into the cell medium for another 12 h of incubation. The adherent cell was washed twice with cold PBS and stained with DAPI, and intracellular fluorescence was observed and captured with a confocal laser scanning microscope (Nikon Ti2) at 60× magnification. In addition, free 5-FAM was set as a control group.

### Cell Protection Examination under Oxidation Stress

BV2 cells, N2a cells, or the BV2–N2a coincubation system (Nanoarchitectonics of tannic acid-based injectable hydrogel regulates the microglial phenotype to enhance neuroplasticity for poststroke rehabilitation) was digested during the logarithmic growth phase and seeded into 96-well cell plates at a density of 1 × 10^5^ cells/well. After cell adhering, the reagent was added according to the manufacturer’s specification (metal-phenolic networks nanoplatform to mimic antioxidant defense system for broad-spectrum radical eliminating and endotoxemia treatment). Free NAC and NNC were further added for another 24 h of incubation, and then CCK-8 solution was added for another 1 h of incubation. The content of water-soluble formazan had a positive correlation with cell viability, which could be detected by its absorption intensity at 450 nm with a multimode reader. In addition, whole cells without treatment were set as 100% cell viability.

### CCK8 Assay

The CCK8 assay was performed using a CCK8 kit following the manufacturer’s protocol. Briefly, BV2 and N2a cells were plated into 96-well plates (5 × 10^3^ cells per well) in 100 μL of culture medium or serum-free condition for 24 h at 37 °C. CCK-8 solution (100 μL/well) was added for another 4 h. Then, the optical density (OD) was measured at 450 nm with a microplate reader (NCM Biotech).

### Mice and Animal Ethics

All mice were individually housed and bred in the Comparative Medicine animal facilities of the Tongji University School of Medicine. All procedures were conducted according to protocols approved by the Institutional Animal Care and Use Committee of the Tongji University School of Medicine.

### Primary Cell Culture

Primary microglia culture: Primary microglia were isolated according to previously published procedure.^41^ In brief, on postnatal day 1, mouse brains were dissected and digested in 0.25% trypsin–EDTA (Gibco) supplemented with 0.05% DNase I at 37 °C for 30 min. After digestion stopped by FBS, tissue precipitation was centrifuged at 1500 rpm at 4 °C for 5 min, resuspended in culture medium, which consisted of DMEM (Gibco) supplemented with 10 ng/mL GM-CSF and 10% FBS, 50 U penicillin, and 50 mg/mL streptomycin at 37 °C, and then filtered through a 40 μm sieve. In addition, culture dishes were coated with 100 mg/mL poly-d-lysine (Sigma) and 5 mg/mL fibronectin (Sigma) for culturing the above microglia-mixed cells, and the medium was replaced every 3 days. Mouse primary microglia in the microglia-mixed cultures were induced to detach by shaking. Floating microglia were collected by centrifugation at 4 °C for 5 min.

Primary neuronal culture: The mouse brain was dissected at embryonic day 16.5 and placed in digestion solution containing 0.25% trypsin–EDTA. After digestion was stopped by FBS, the tissue was triturated 12 times using the side of a pipet until no large pieces of tissue remained. The dissociated tissue was centrifuged at 1500 rpm for 5 min, and the cell pellet was resuspended in Neurobasal medium (Thermo Fisher Scientific) containing 2% B27 Supplementary (Thermo Fisher Scientific), 1% GlutaMAX (Thermo Fisher Scientific), 50 U penicillin, and 50 mg/mL streptomycin. Culture dishes were coated with 100 mg/mL poly-d-lysine (Sigma) the night before use. Cells were cultured in a humidified atmosphere containing 5% CO_2_ at 37 °C, with medium half-changes every 3 days.

### Cell Supernatant Fluorescence Detection Assay

In order to further detect the uptake and release of TLQP21 in the TNNC by different neurological cells under oxidative stress, we labeled TLQP21 with 5-FAM fluorescent dye in the TNNC and detected the cell supernatant and lysate via the fluorescent microplate reader. The primary cultured neurons and microglia plated in the 12-well plates were treated with different concentrations of 3% H_2_O_2_ for 4 h to stimulate neurons to generate oxidative stress. After that, the original medium was discarded, and the fresh medium mixed with 1 mg/mL TNNC-FAM was incubate for 24 h. To exclude the interference of the original TNNC-FAM, we discarded the supernatant of the medium after 24 h and collected the cell supernatant in the next 2 h. After removing the cell supernatant, cell lysis buffer was added to each well in the 12-well plate. We collected the cell lysis supernatant via centrifugation and added 100 μL of collected cell lysis supernatant and cell supernatant into each well of the 96-well plate. Finally, the fluorescence intensity was detected by a fluorescent microplate reader, with excitation light at 485 nm and emission light at 535 nm.

### RNA Isolation and Library Preparation

RNA isolation and library preparation: The TRIzol reagent (Invitrogen, CA, USA) was utilized for the extraction of total RNA in accordance with the guidelines provided by the manufacturer. To determine the RNA purity and concentration, a NanoDrop 2000 spectrophotometer (Thermo Scientific, USA) was employed. Evaluation of RNA integrity was performed using an Agilent 2100 Bioanalyzer (Agilent Technologies, Santa Clara, CA, USA). Subsequently, libraries were generated following the manufacturer’s guidelines with the VAHTS Universal V6 RNA-seq Library Prep Kit. The transcriptome sequencing and subsequent analysis were carried out by OE Biotech Co., Ltd. (Shanghai, China).

### mRNA Sequencing Analysis Process

Libraries were sequenced by using the Illumina NovaSeq 6000 platform, resulting in the generation of 150 bp paired-end reads. Each sample produced approximately 46.97 M raw reads. The raw FASTQ reads were first processed with fastp,^42^ allowing for the removal of low-quality sequences and leading to the acquisition of clean reads. Subsequently, around 46.51 M clean reads per sample were retained for further analysis. These clean reads were then aligned to the *Mus musculus* genome utilizing HISAT.^43^ FPKM^44^ values for each gene were computed, while read counts for the genes were determined via HTSeq-count.^45^ Principal component analysis (PCA) was conducted using R (v 3.2.0) to assess the biological variation among samples.

For the differential expression analysis, DESeq2 ^46^ was employed, setting a threshold of *p*-value <0.5 or fold change >2 to identify significantly differentially expressed genes (DEGs). Hierarchical clustering of DEGs was executed using R (v 3.2.0) to illustrate gene expression patterns across various groups and samples. A radar plot was created using the R package ggradar to display the expression levels of either upregulated or downregulated DEGs. Additionally, pathway enrichment analysis such as Gene Ontology (GO)^47^ and KEGG^48^ were conducted on DEGs based on hypergeometric distribution to identify significantly enriched terms, utilizing R (v 3.2.0) for these analyses. R (v 3.2.0) was also employed to create column diagrams, chord diagrams, and bubble diagrams representing the significant enrichment terms. Gene Set Enrichment Analysis (GSEA) was carried out using GSEA software.^49,50^ This analysis utilized a predefined gene set, ranking the genes according to their differential expression levels between the two sample types, and examined whether the predefined gene set was enriched at the top or bottom of the ranked list.

### Measurement of Microglia ROS Production

The staining procedure was performed using a working solution prepared by diluting the stock solution in PBS or serum-free culture medium to a final concentration of 5 μM (1:1000).

Cells were seeded into 24-well plates at a density of 2 × 10^5^ cells per well. A volume of 1 mL of the working solution was added to each well, and the plate was incubated under light-protected conditions for 30 min at 37 °C. After incubation, the staining solution was aspirated, and the cells were rinsed twice with 1× PBS to remove any residual dye. Stained cells were then maintained in PBS and immediately imaged using a fluorescence microscope to assess the staining.

### Microglia Phagocytosis Assay

As previously described,^51^ fluorescent-labeled latex bead suspensions (Sigma) were mixed with FBS at a volume ratio of 1:5 for 1 h. Microglia culture medium was added to dilute the medium to reach the final content of beads (0.01% *v*/*v*) and FBS (0.05% *v*/*v*). After uridine diphosphate (UDP) was added at a final concentration of 100 μM for 1 h of incubation, the fluorescence signal was recorded and located by a living image device. The percentage of phagocytic cells was determined by counting the number of microglia with intracellular fluorescence.

### Transwell Assay

Primary microglia were plated onto a Transwell insert (8.0 μm pores, 24-well, Corning). A 1 mM concentration of adenosine triphosphate (ATP) was added to the bottom well to stimulate primary microglia downward migration. After 24 h of incubation, all cells in the bottom well were fixed with freshly prepared 4% paraformaldehyde (PFA) for 15 min, stained with 0.1% crystal violet for 15 min, and then imaged with an inverted microscope. The cells on the upper surface were removed with cotton swabs. The mean number of migrated cells was determined with ten fields for each insert per experiment.

### Chronic Unpredictable Mild Stress (CUMS) Model Building

C57BL/6 mice were single-bred and received 3 weeks of unpredictable mild stressors. All of the stressors were performed randomly within 1 week. Such stressors consisted of (i) intermittent illumination (24 h), (ii) 45° cage tilting (24 h), (iii) damp bedding (24 h), (iv) food and water deprivation (24 h), (v) reverse light/dark cycle, (vi) restraint in a 50 mL tube (4 h), and (vii) noise interference (12 h). Mice grouped in a neutral environment were set as the control group. The stress procedure continued for 3 weeks prior to behavioral tests, and various behavioral tests were performed for examining the CUMS model build.

### Behavioral Analysis

Sucrose preference test: Each mouse was adapted to two bottles of 2% sucrose for 24 h and then given access to 2% sucrose and water for 12 h to evaluate the baseline of each mouse. Mice were deprived of food and water for 24 h before the test. On test day, each mouse was given one bottle of water (B) and one bottle of 2% sucrose (A) for 1 h and switched the bottle position to avoid a side bias. Related volume (Vol) reduction was counted after 24 h of experiment, and the percentage of sucrose preference was calculated using the formula 100*­[Vol_A_/(Vol_A_ + Vol_B_)].

Tail suspension test: The tail of each mouse was tied 25 cm away from the table. Mice were regarded as being in the immobile condition when they abandoned struggling and were completely still. The study lasted 6 min, involving 2 min of adapted times and 4 min of test times, and the percentage of immobility condition and global activities were analyzed by SMART software (RWD Life Science).

Open field test: Mice were placed into the center of an open field area (40 × 40 × 40 cm) with a black bottom and walls and then allowed to freely explore for 6 min. The distance traveled by mice was recorded during the last 5 min and analyzed by SMART software (RWD Life Science). Additionally, the chamber was cleaned with 75% ethyl alcohol to avoid the effect of smell on the next test subject.

Forced swimming test: Each mouse was placed for 6 min in a cylinder (height: 30 cm, diameter: 20 cm) containing 15 cm of water. The duration of immobility was measured during the last 4 min of swimming time by SMART software (RWD Life Science).

### Stereotactic Injection

Three-month-old CUMS mice were anesthetized, and drugs (Supporting Information Table 1) or PBS was injected into the hippocampi using an automated stereotaxic injection apparatus (RWD Life Science, Shenzhen, China) at bilateral hippocampi coordinates: anteroposterior, −1.7 mm from the bregma; mediolateral, ± 2.0 mm; dorsoventral, −1.75 mm. Following all surgical procedures, animals recovered on a heating pad for 24 h of postsurgery recovery and monitoring and then returned to their home cages.

### Au Nanocage Pharmacokinetics

The quantification of gold in brain tissue samples was performed using an Agilent 7700 inductively coupled plasma mass spectrometry (ICP–MS) system. Following a standardized microwave-assisted acid digestion protocol with RIPA lysis buffer, the digested samples were diluted and introduced to the ICP–MS via a microflow PFA nebulizer and a Peltier-cooled cyclonic spray chamber. The sample introduction system was set with an uptake time of 45 s and a stabilization time of 30 s to ensure signal stability. An integration time of 0.90 s per mass was used for data acquisition. Internal standardization was employed throughout the analysis to correct for signal drift and matrix effects, ensuring robust and accurate quantification of gold content.

### Western Blotting

Tissues or cells were lysed in RIPA lysis buffer and extraction buffer (Thermo Fisher Scientific) for quantification of protein content as previously described.^52^ Related protein concentrations were determined by a BCA Protein Assay Kit (Pierce) and then separated by sodium dodecyl sulfate-polyacrylamide gel electrophoresis (SDS-PAGE) and transferred to a hydrophobic PVDF transfer membrane, and corresponding membranes were blocked with 5% no-fat milk subsequently. The membranes were successively incubated with primary antibodies and HRP-conjugated secondary antibodies diluted in blocking solutions and washed 3 times with TBST for 10 min each. Bound antibodies were visualized by Pierce ECL Western Blotting Substrate (Thermo Fisher). For data quantification, films were scanned with a CanonScan 9950F scanner, and the acquired images were analyzed by the ImageJ program.

### Quantitative Reverse Transcription-Polymerase Chain Reaction (RT-qPCR)

Total RNA was isolated from cell or tissue samples by the FastPure Cell/Tissue total RNA isolation kit (Nanjing Vazyme Biotech Co., Ltd.) according to the manufacturer’s instructions, and its quantity and quality were assessed using NanoDrop ND1000 (Thermo Fisher Scientific, Waltham, MA).

Equal amounts of mRNA templates were used for complementary DNA synthesis using HiScript III All-in-one RT SuperMix (Vazyme). The transcripts were amplified by gene-specific primers (Supporting Information Table 2) and the SYBR green PCR Kit (Vazyme) with the ABI7500 (Applied Biosystems). Each sample was measured in triplicate for all RT-qPCR outcomes, and negative controls were used with no template. Amplification curves and gene expression were standardized against Gapdh (for mRNA).

### TUNEL Assay

Brain tissues were sliced using a frozen slicer (Leica) at a thickness of 20 μm. Frozen slices were placed on a long rack, let dry at RT for 20 min, and then submerged in 4% PFA at RT for 30 min. Terminal deoxynucleotidyl transferase-mediated dUTP nick end labeling (TUNEL) assay was then performed using the TUNEL assay kit (Vazyme, A111-01) according to the manufacturer’s instruction. Images were taken by an Olympus confocal microscope FV 3000. Stained brain slices were then used to quantify the fluorescence intensity of TUNEL via ImageJ.

### Immunofluorescence

Brain tissues were sliced using a freezing slicer (Leica) at a thickness of 20 μm. The fixation of tissue sections for frozen sections was carried out using 4% PFA. The samples were subsequently permeabilized with 0.3% Triton-X, followed by blocking using 10% goat/donkey serum and 0.5% Tween-20 1× PBS solution. The sections were then incubated with specific primary antibodies (Supporting Information Table 3) overnight at 4 °C. The slides were subsequently incubated with Alexa Fluor-conjugated secondary antibodies for 1 h at RT and mounted in Vectashield mounting medium containing DAPI. Finally, the immunofluorescence images were captured using an Olympus confocal microscope FV 3000.

### Golgi Staining

Golgi staining was performed as previously described.^53^ In brief, mouse brains were carefully dissected and washed in Milli-Q water to remove blood from the surface. The brains were immersed in a mixture of FD solution A + B (FD Neurotechnologies Inc., FD Rapid Golgistain Kit, PK401C) at a volume ratio of 1:1, stored in the dark at RT for 14 days, and then incubated in FD solution C at RT in the dark for 7 days. Brain tissues were sliced using a freezing slicer (Leica) at a thickness of 100 μm. Each brain slice was mounted on gelatin-coated microscope slides with solution C and dried naturally at RT. Sections were washed in ddH_2_O twice and then placed in a mixture of FD solution D/E/ddH_2_O at a volume ratio of 1:1:2 for 10 min. Subsequently, all samples were subjected to a dehydration process with 50%, 75%, 95%, and 100% ethanol, each for a duration of 4 min. Finally, the sections were sealed with an antifluorescent quenching agent, cleared in xylene thrice, and photographed with an Olympus confocal microscope FV 3000.

### Flow Cytometry

Flow cytometry was used to detect the ROS levels. For cell samples, neurons were treated with 0, 100, and 200 μM H_2_O_2_ and incubated for 4 h. After harvesting, cells were washed with PBS to remove any remaining cellular debris and stained with red fluorescent dye for 30 min at 37 °C. After staining, cells were washed again with PBS to remove any unbound antibodies and finally resuspended in a small volume of PBS for flow cytometry analysis. For tissue samples, brain tissues were digested into a single-cell suspension, washed with PBS to remove any remaining cellular debris, stained with 10 μM DCFH-DA fluorochrome for 30 min in the dark, and immediately taken to the Flow Cytometry core facility. At least 5000 cells were collected in the ROS-specific fluorescent dye gate and analyzed by flow cytometry.

### Patch-Clamp Recordings from the Hippocampal CA1 Region

For spontaneous excitatory postsynaptic current (EPSC) measurements, 3 month-old CUMS mice were used. Heads were decapitated under anesthesia with isoflurane. Brains were rapidly dissected and transferred to ice-cold oxygenated sucrose-substituted artificial cerebrospinal fluid (aCSF) solution, composed of (in mM) 125 NaCl, 1.25 NaH_2_PO_4_, 2.5 CaCl_2_, 2.5 KCl, 1.3 MgCl_2_, 25 NaHCO_3_, and 10 d-glucose. Coronal brain slices (300 μm thickness) were prepared with a Vibratome (Leica VT1200S) in oxygenated cold dissection buffer containing 212 mM sucrose, 25 mM NaHCO_3_, 5 mM KCl, 1.25 mM NaH_2_PO_4_, 0.5 mM CaCl_2_, 3.5 mM MgSO_4_, 10 mM d-glucose, 1.25 mM l-ascorbic acid, and 2 mM Na-pyruvate. Slices were transferred into an incubation chamber with normal aCSF and bubbled with 95% O_2_/5% CO_2_. After 20 min of recovery at 32 °C, slices were incubated for at least 1 h at RT (25 ± 2 °C) prior to electrophysiological studies. A patch of membrane was subsequently ruptured by mild suction and monitored for 3 min. EPSCs (by holding at −70 mV) were then recorded in sequence.

### Statistical Analysis

Normal distribution was assessed using the Shapiro–Wilk test. The statistical difference between two independent groups was analyzed with the unpaired Student’s *t*-test, and that among more than two groups was assessed with the parametric one-way ANOVA with Tukey’s multiple comparisons test. Data were shown as the mean ± s.d., and significance was determined as *p* < 0.05.

## Supplementary Material



## Notes

Biosafety of Au nanocages (AuNCs); dose-dependent effects of H_2_O_2_ on ROS levels in primary neurons; TNNC alleviation of H_2_O_2_-induced inflammatory responses of BV2 cells; TNNC repression of inflammatory protein expression in H_2_O_2_-exposed N2a cells; CUMS-induced depressive behaviors; CUMS-induced ROS accumulation, synaptic vesicle reduction, and disorganized mitochondrial cristae in mouse brains; Cox2 was highly expressed in neurons; validation of gene expression in the RNA-seq; TNNC inhibited CUMS-induced astrocytic activation; C1qR downstream P2RY6 and P2RY12 modulation of microglial migration and phagocytosis; C3aR modulated inflammatory responses of microglia; experimental design of TNNC treatment of CUMS mice (groups, dosages, and animal numbers); primer sequences for RT-qPCR analysis; and reagent information (PDF)
