# Supplementary material for: N‑Acetylcysteine-Capped TLQP21-Containing Au Nanocages Alleviate Depression in Mice
Source: ACS Nano. 2025 Oct 14;19(42):37186–205. doi: 10.1021/acsnano.5c11681 (PMC12574220; doi:10.1021/acsnano.5c11681)
Supplement: Supplementary file 1 [file nn5c11681_si_001.pdf]

Supplementary Information:

N-acetylcysteine-Capped TLQP21-Containing Au Nanocages Alleviate Depression in Mice

Meng Shi[1ǂ], Xiangyu Li[1ǂ], Zhen Fan[2ǂ], Yi Wang[1], Congcong Li[1], Yuanmeng Ning[1], Yizhao Ma[1], Min Sun[1, 3*], Xiaohuan Xia[1, 3*], Jianzhong Du[1, 3*], and Jialin C. Zheng[1, 3, 4*]

[1]Center for Translational Neurodegeneration and Regenerative Therapy, Tongji Hospital affiliated to Tongji University School of Medicine, Shanghai 200065, China.

[2]Shanghai Key Laboratory of Anesthesiology and Brain Functional Modulation, Clinical Research Center for Anesthesiology and Perioperative Medicine, Translational Research Institute of Brain and Brain-Like Intelligence, Shanghai Fourth People's Hospital, School of Medicine, Tongji University, Shanghai 200434, China.

[3]Department of Polymeric Materials, School of Materials Science and Engineering, Tongji University, Shanghai 201804, China.

[4]Translational Research Center, Shanghai Yangzhi Rehabilitation Hospital affiliated to Tongji University School of Medicine, Shanghai 201619, China;

[5]School of Materials Science and Engineering, East China University of Science and Technology, 130 Meilong Road, Shanghai 200237, China

[6]State Key Laboratory of Cardiology and Medical Innovation Center, Shanghai East Hospital, School of Medicine, Tongji University, Shanghai 200120, China.

[7]Innovation center of medical basic research for brain aging and associated diseases, Ministry of Education, Tongji University, Shanghai 200330, China.

[8]Collaborative Innovation Center for Brain Science, Tongji University, Shanghai 200072, China.

[ǂ]These authors contributed equally to this work

[*]Corresponding authors: Drs. Jialin C. Zheng, Jianzhong Du, Xiaohuan Xia, Min Sun

Email: jialinzheng@tongji.edu.cn; jzdu@tongji.edu.cn; xiaohuan_xia1@163.com; 22310108@tongji.edu.cn.

Contents

Supplementary Figures S1-S11

Supplementary Tables S1-S3

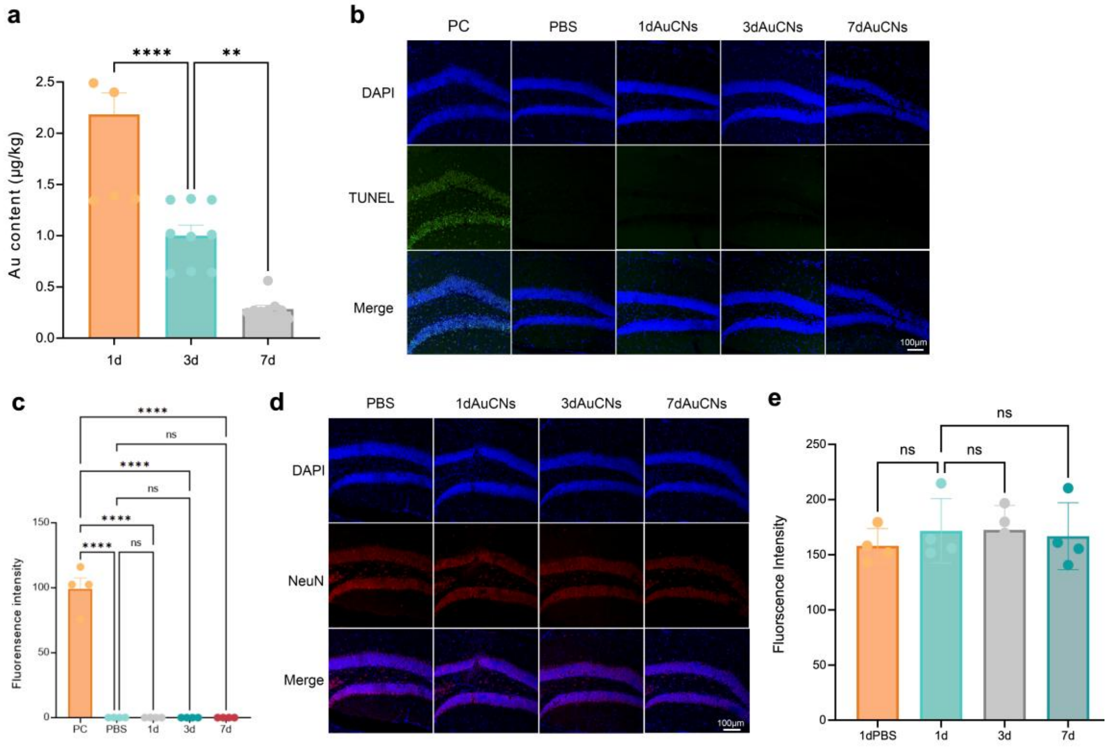

Figure S1 | The biosafety of Au nanocages (AuNCs).

a, Detection of Au content in brain tissue lysate at different time points after stereotactic injection of AuNCs. **b, c,** The fluorescence intensity of TUNEL staining was detected the apoptotic cells after stereotactic injection of AuNCs into the brain (n = 4, one-way ANOVA), Scale bar 100μm. **d, e,** The fluorescence intensity of $NeuN^+$ neurons was detected at different time points after the stereotactic injection of AuNCs into the brain by immunofluorescence (n = 4, one-way ANOVA), Scale bar 100μm. All data are represented as means ± s.d. ****$p < 0.0001$, **$p < 0.01$, and ns represents non-significant.

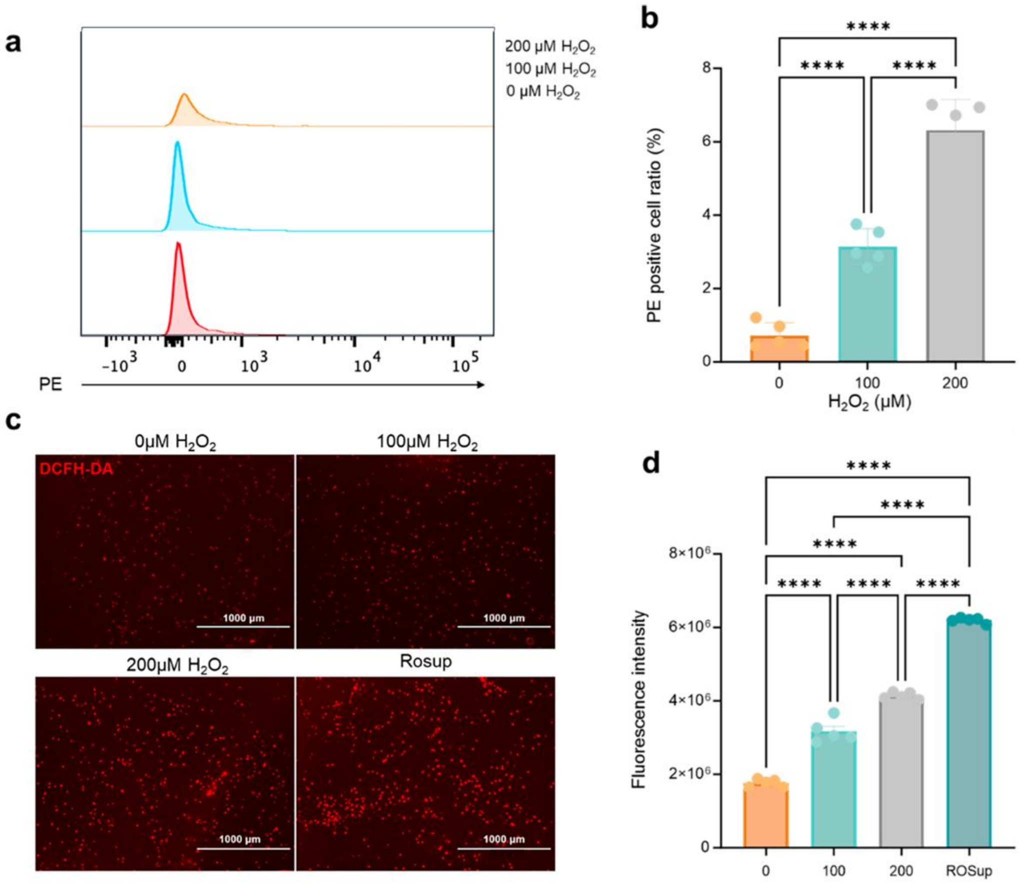

Figure S2 | Dose dependent effects of H_2O_2 on ROS levels in primary neurons.

a, Flow cytometry detects for primary neuron total ROS in different H_2O_2 concentrations. **b,** Quantitative comparison of cellular ROS levels between 0, 100, and 200 μM H_2O_2 (n = 5, one-way ANOVA). All data are represented as means ± s.d. ****$p < 0.0001$.

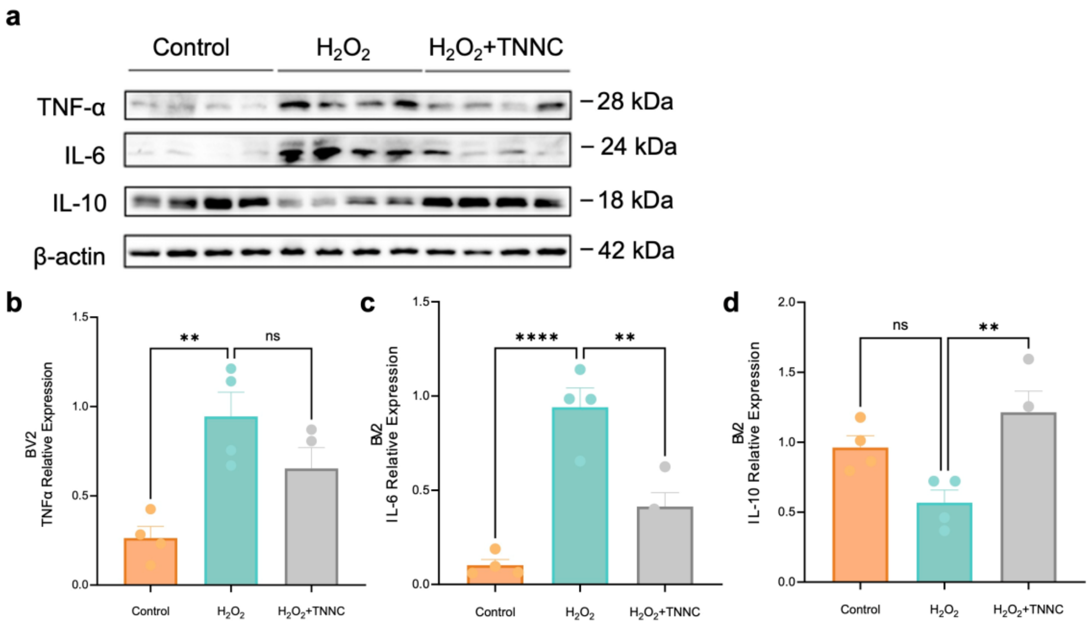

Figure S3 | TNNC alleviated H_2O_2-induced inflammatory responses of BV2 cells.

a, The levels of inflammation-related proteins TNF-α, IL-6, and IL-10 in H_2O_2-exposed BV2 cells with/without TNNC treatment were determined by western blotting. **b-d**, Quantitative results of TNF-α (**b**), IL-6 (**c**), and IL-10 (**d**) protein levels (n = 4, one-way ANOVA). All data are represented as means ± s.d. ****$p < 0.0001$, **$p < 0.01$, and ns represents non-significant.

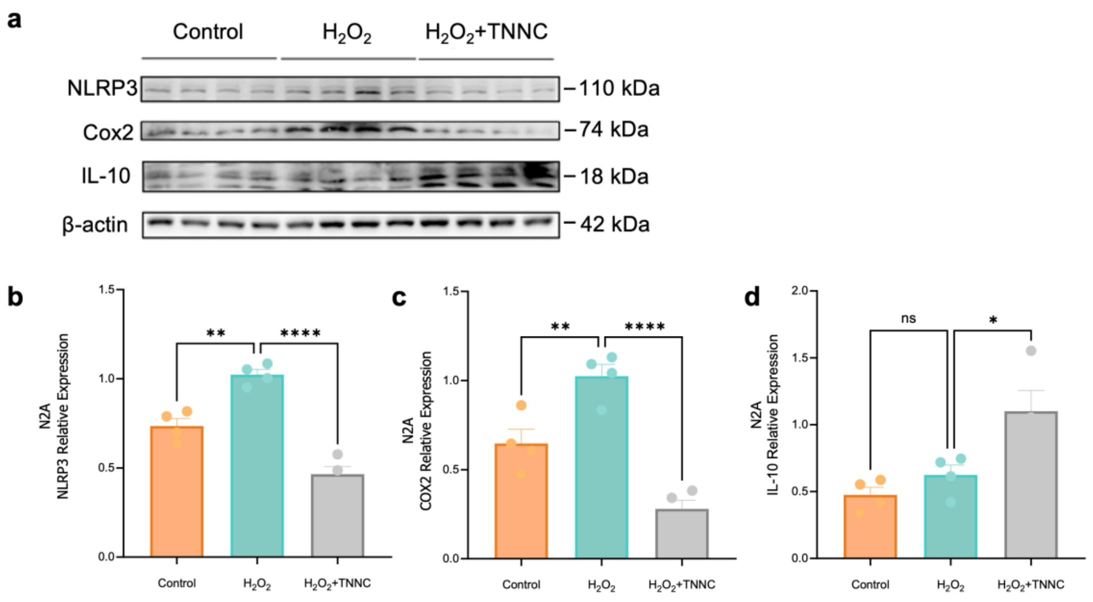

Figure S4 | TNNC repressed inflammatory protein expression in H_2O_2-exposed N2a cells. **a,** The levels of inflammation-related proteins NLRP3, Cox2, and IL-10 in H_2O_2-exposed N2a cells with/without TNNC treatment were determined by western blotting. **b-d**, Quantitative results of NLRP3 (**b**), Cox2 (**c**), and IL-10 (**d**) protein levels (n = 4, one-way ANOVA). All data are represented as means ± s.d. ****$p < 0.0001$, **$p < 0.01$, *$p < 0.05$, and ns represents non-significant.

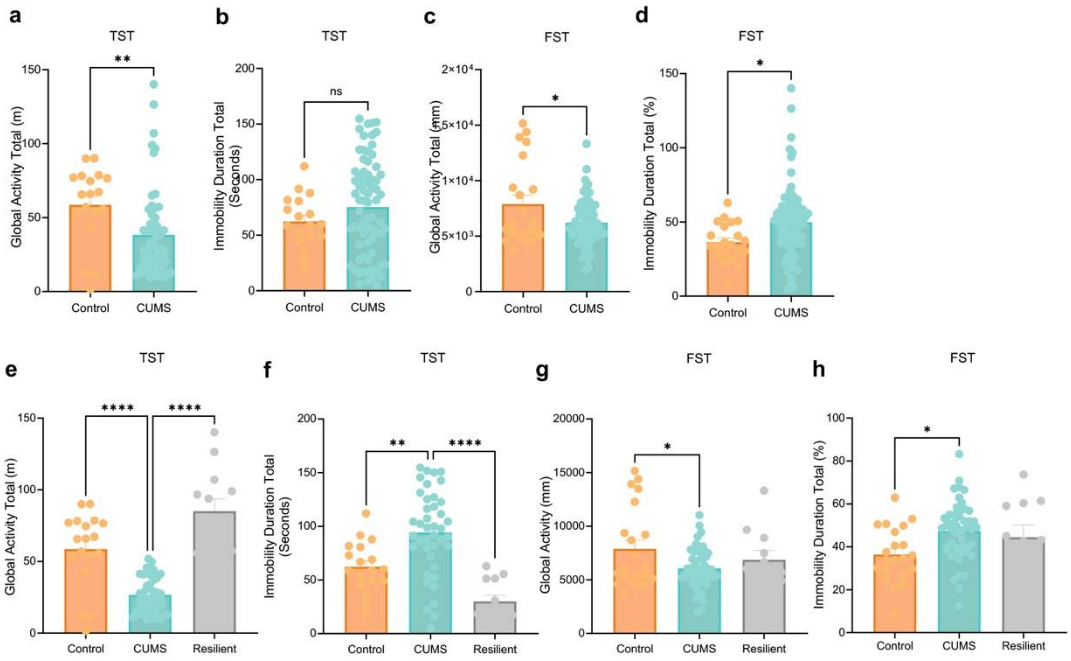

Figure S5 | CUMS induced depressive behaviors.

a, Tail suspension test: global activity (Control n = 18, CUMS n = 60, one-way ANOVA). **b,** Tail suspension test: immobility time (Control n = 18, CUMS n = 60, one-way ANOVA). **c,** Forced swimming test: global activity (Control n = 18, CUMS n = 60, one-way ANOVA). **d,** Forced swimming test: percentage of immobility condition (Control n = 18, CUMS n = 60, one-way ANOVA). **e,** Tail suspension test: global activity (Control n = 18, CUMS n = 48, Resilient n = 12, one-way ANOVA). **f,** Tail suspension test: immobility time (Control n = 18, CUMS n = 48, Resilient n = 12, one-way ANOVA). **g,** Forced swimming test: global activity (Control n = 18, CUMS n = 48, Resilient n = 12, one-way ANOVA). **h,** Forced swimming test: percentage of immobility condition (Control n = 18, CUMS n = 48, Resilient n = 12, one-way ANOVA). All data are represented as means ± s.d. ****$p < 0.0001$, **$p < 0.01$, *$p < 0.05$, and ns represents non-significant.

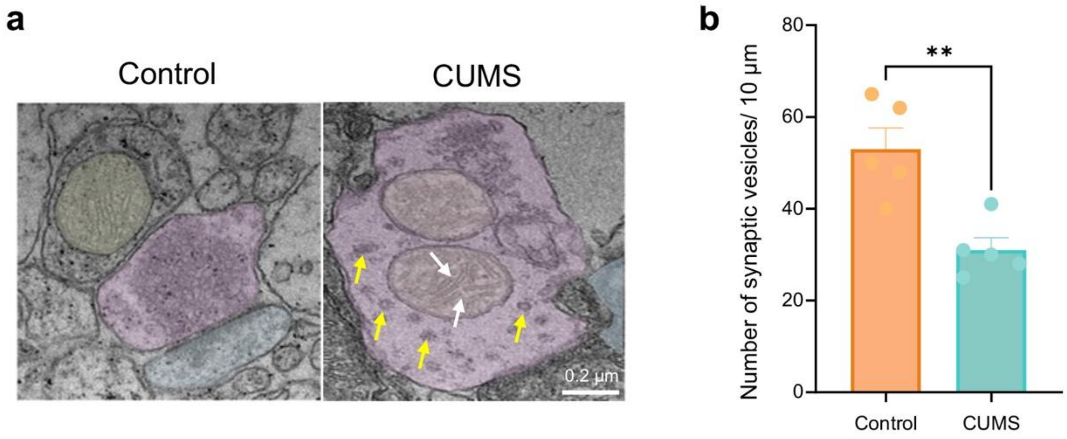

Figure S6 | CUMS induced ROS accumulation, synaptic vesicle reduction, and mitochondrial cristae disorganized in mouse brains.

a, Representative electron microscopic image of hippocampi showing that CUMS mice present Presynaptic membrane swelling, synaptic vesicles significantly reduced (yellow arrow) and mitochondrial cristae disorganized (white arrow). Scale bar, 0.2 μm. **b,** Quantification results of synaptic vesicle number of Control and CUMS (n = 5, unpaired *t*-test). All data are represented as means ± s.d. $^{**}p < 0.01$.

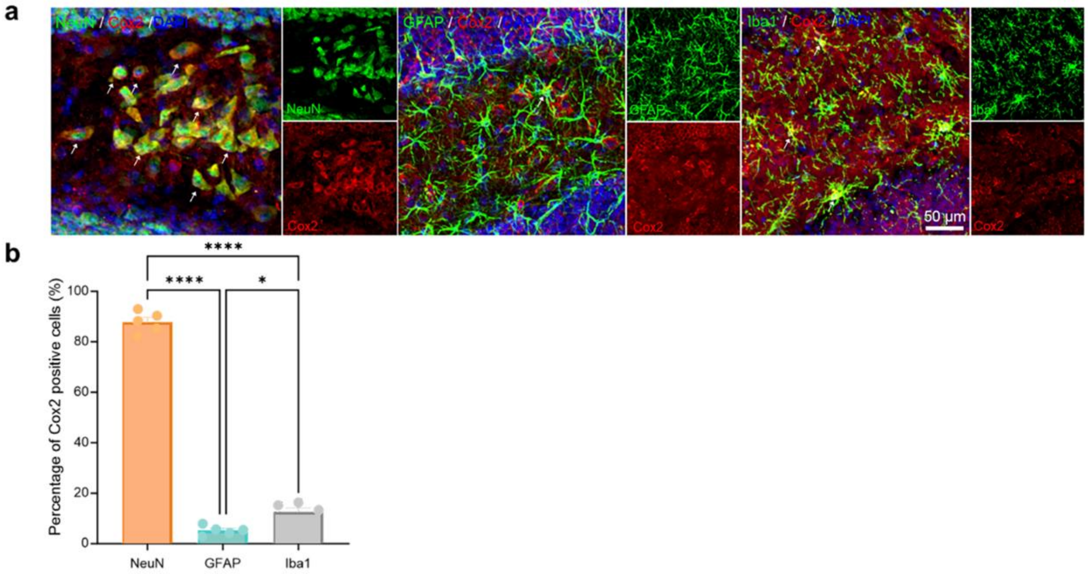

Figure S7 | Cox2 was highly expressed in neurons.

a, Schematic diagram of colocalization of Cox2 (red) with NeuN, GFAP, and Iba1 stained by immunofluorescence, Scale bar 50 μm. **b,** Quantification results of percentage of Cox2 positive cells (n = 5, one-way ANOVA). All data are represented as means ± s.d. ****$p < 0.0001$ and *$p < 0.05$.

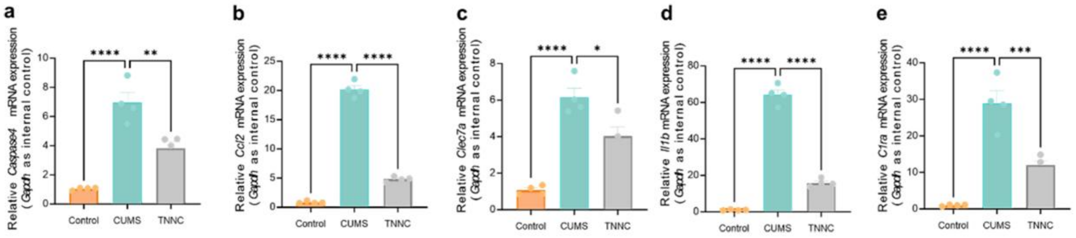

Figure S8 | Validation of gene expression in the RNA-seq.

a-e, RT-qPCR analyses of *Caspase4* (**a**), *Ccl2* (**b**), *Clec7a* (**c**), *Il1b* (**d**), *C1ra* (**e**) transcript levels in WT mice, CUMS mice, and CUMS mice treated by TNNC (n=4, one-way ANOVA). All data are represented as means ± s.d. ****$p < 0.0001$, ***$p < 0.001$, **$p < 0.01$, and *$p < 0.05$.

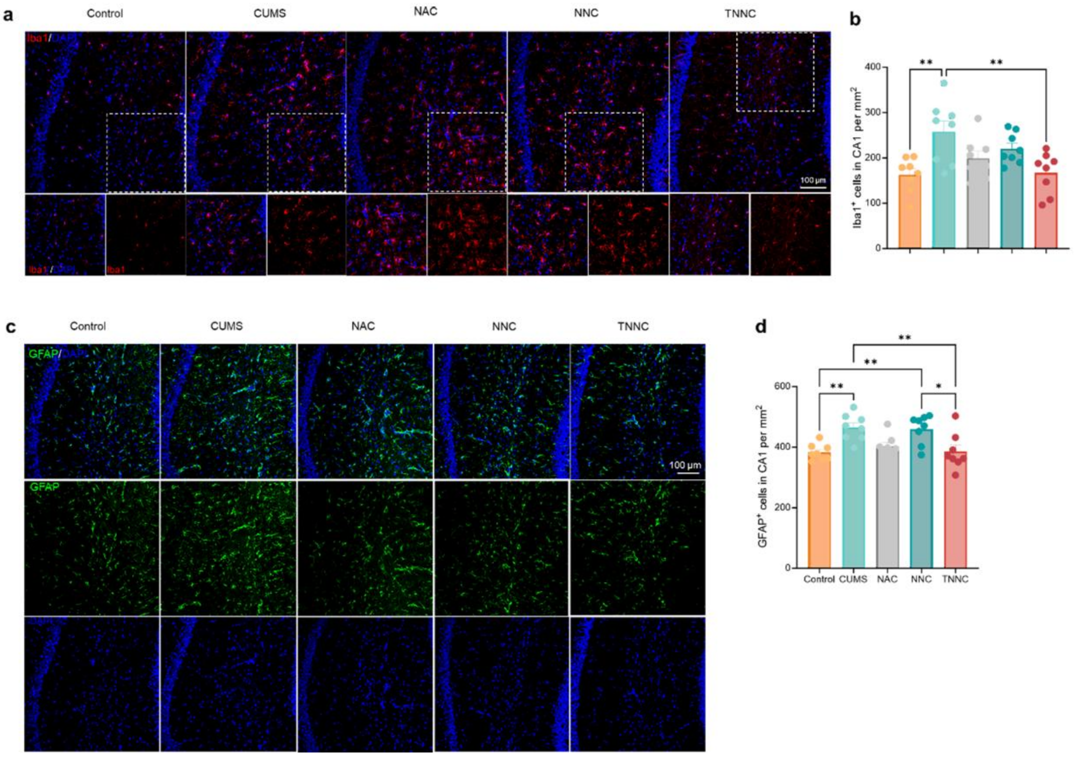

Figure S9 | TNNC inhibited CUMS-induced astrocytic activation.

a, Representative confocal microscopic images of immunostainings for and Iba1$^+$ cells in CA1 regions of the hippocampi. Scale bar 100 µm. **b,** Quantitative results of Iba1$^+$ cell numbers in the CA1 region of the hippocampi (n = 8, one-way ANOVA). **c,** Representative confocal microscopic images of immunostainings for and GFAP$^+$ cells in CA1 regions of hippocampi. Scale bar 100 µm. **d,** Quantitative results of GFAP$^+$ cell numbers in the CA1 region of the hippocampi (n = 8, one-way ANOVA). All data are represented as means ± s.d. **$p < 0.01$ and *$p < 0.05$.

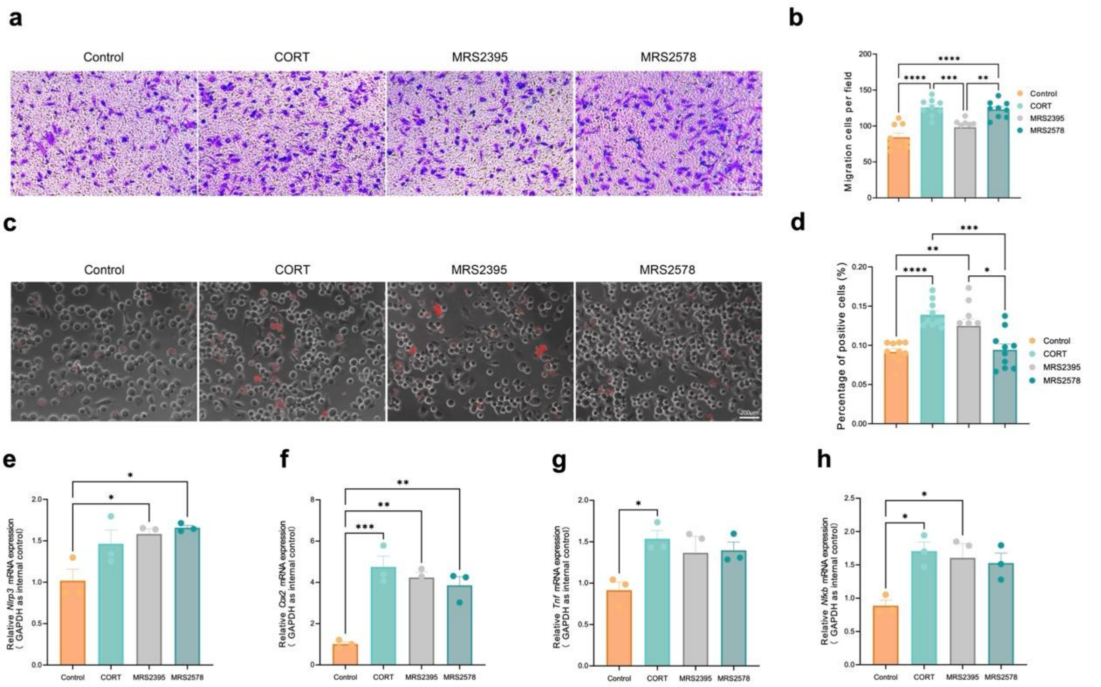

Figure S10 | C1qR downstream P2RY6 and P2RY12 modulated microglial migration and phagocytosis.

a, Representative images of migrated microglia treated with CORT, P2Y12 inhibitor MRS2395, and P2RY6 inhibitor MRS2578. Scale bar 100 μm. **b,** Quantification of migrated cell numbers of each group under chamber membrane (n = 10, one-way ANOVA). ATP (1 mM) is used as a chemoattractant. **c,** Phagocytosis assay *in situ.* Representative images of microglia treated with CORT, P2Y12 inhibitor MRS2395, and P2RY6 inhibitor MRS2578 after 60 mins incubation with latex beads. Scale bar 200 μm. **d,** Quantification of the proportions of microglia containing beads in different groups (n = 10, one-way ANOVA). **e-h**, Quantification of the transcriptional levels of *Nlrp3* (**e**), *Cox2* (**f**), *Tnf* (**g**), and *Nfkb* (**h**) in microglia treated with CORT, P2Y12 inhibitor MRS2395, and P2RY6 inhibitor MRS2578 by RT-qPCR (n = 3, one-way ANOVA). All data are represented as means ± s.d. ****$p < 0.0001$, ***$p < 0.001$, **$p < 0.01$, *$p < 0.05$, and ns represents non-significant.

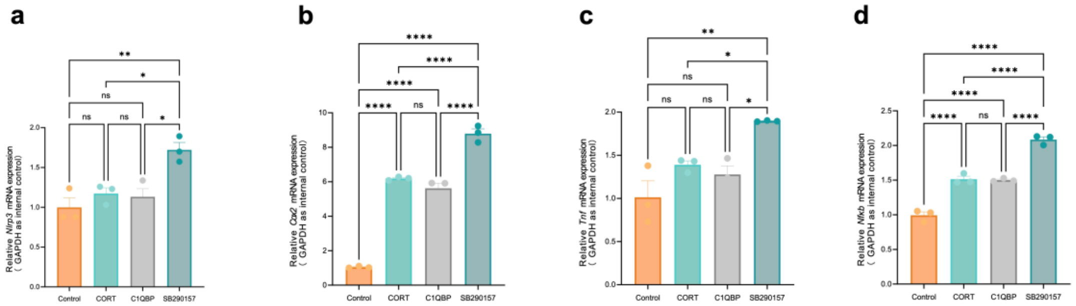

Figure S11 | C3aR modulated inflammatory responses of microglia.

a-d, Quantification of the transcriptional levels of *Nlrp3* (**a**), *Cox2* (**b**), *Tnf* (**c**), and *Nfkb* (**d**) in the microglia treated with CORT, C1QBP, and C3aR1 antagonist SB290157 by RT-qPCR (n = 3, one-way ANOVA). All data are represented as means ± s.d. ****$p < 0.0001$, **$p < 0.01$, *$p < 0.05$, and ns represents non-significant.

Table S1. Experimental design of TNNC treatment of CUMS mice (groups, dosages, and animal numbers).

Group	Material	Subjects	Modes	Dosage	Frequency	Number
**Control**	PBS	Control mice	Stereotype injection	/	/	12
**CUMS**	PBS	CUMS mice	Stereotype injection	/	/	12
**NAC**	NAC	CUMS mice	Stereotype injection	1 mg/mL	Once	12
**NNC**	NAC, AU Nanocages	CUMS mice	Stereotype injection	1 mg/mL	Once	12
**TNNC**	NAC, AU Nanocages, TLQP21	CUMS mice	Stereotype injection	1 mg/mL	Once	12

Table S2. The primer sequences for RT-qPCR analysis.

**List of oligonucleotide sequences**	**5'>3'**
Gapdh (mouse)-F	AACTTTGGCATTGTGGAAGG
Gapdh (mouse)-R	ACACATTGGGGGTAGGAACA
Il1b (mouse)-F	GAAATGCCACCTTTTGACAGTG
Il1b (mouse)-R	TGGATGCTCTCATCAGGACAG
Il6 (mouse)-F	CTGCAAGAGACTTCCATCCAG
Il6 (mouse)-R	AGTGGTATAGACAGGTCTGTTGG
Tnf (mouse)-F	CCTGTAGCCCACGTCGTAG
Tnf (mouse)-R	GGGAGTAGACAAGGTACAACCC
Il4 (mouse)-F	GGTCTCAACCCCCAGCTAGT
Il4 (mouse)-R	GCCGATGATCTCTCTCAAGTGAT
Il10 (mouse)-F	CTTACTGACTGGCATGAGGATCA
Il10 (mouse)-R	GCAGCTCTAGGAGCATGTGG
Nlrp3 (mouse)-F	ATTACCCGCCCGAGAAAGG
NLlrp3 (mouse)-R	CATGAGTGTGGCTAGATCCAAG
Bax (mouse)-F	AGACAGGGGCCTTTTTGCTAC
Bax(mouse)-R	AATTCGCCGGAGACACTCG
Tp53 (mouse)-F	CCCCTGTCATCTTTTGTCCCT
Tp53 (mouse)-R	AGCTGGCAGAATAGCTTATTGAG
Caspase3 (mouse)-F	CTCGCTCTGGTACGGATGTG
Caspase3 (mouse)-R	TCCCATAAATGACCCCTTCATCA

Table S3. Reagent informations

Reagent	Source	Identifier
Antibodies		
C1qbp	Proteintech	24474-1-AP
GFAP	CST	80788
GFAP	Sigma	G3893
PSD95	CST	3409
Synaptophysin	CST	36406
NLRP3	SantaCruz	134306
NF-κB	CST	9936T
NeuN	Millipore	MAB377B
GPX4	Abclonal	A21440
Cox2	Abcam	Ab15191
β-Actin	Sigma	A5441
Iba1	Abcam	Ab283346
Iba1	SYSY	234009
HPR-Goat anti Rabbit Antibody	Icllab	GGHL-15P
HPR-Goat anti Mouse Antibody	Icllab	GGHL-90P
Alexa Fluor488, 568, 647 Goat anti Rabbit	Life Technologies	
Alexa Fluor488, 568, 647 Goat anti Mouse	Life Technologies	
Alexa Fluor647 Goat anti Chicken	Life Technologies	
Chemicals, peptides, and recombinant proteins		
Golgi Stain Kit	FD	PK401
Penicillin/ Streptomycin	Gibco	15140-122
Elisa Kit (IL-1β, TNF-α)	LCSciences	70-EK206/3-48, 70EK201B/3-48
5x SDS	Beyotime	P0015L
Tissue lysis buffer	Thermofish	78516
Primer	Tsingke	N/A
Trizol	Vazyme	R401-01
RNA Isolation Kit	Vazyme	RC112-01
All-in-one RT SuperMix	Vazyme	R333-01
SYBR Green	Vazyme	Q712-02
Corticosterone	Selleck	S4752
Fluorescent latex beads	Sigma	L1030
Crystal Violet Ammonium Oxalate	Solarbio	G1063
ROS detection kit	Solarbio	CA1420
Transwell	Corning	CLS3396-2EA
2′, 7′-Dichlorofluorescin diacetate	Solarbio	D6470
3% H_2O_2	Sinopharm chemical reagent	10011218
MRS2395	Sigma	M5942
MRS2578	Sigma	M0319
